# In-line NIR coupled with machine learning to predict mechanical properties and dissolution profile of PLA-Aspirin

**DOI:** 10.1186/s42252-024-00063-5

**Published:** 2024-10-08

**Authors:** Nimra Munir, Tielidy de Lima, Michael Nugent, Marion McAfee

**Affiliations:** 1 Centre for Mathematical Modelling and Intelligent Systems for Health and Environment (MISHE), Atlantic Technological University, ATU Sligo, Ash Lane, Co. Sligo F91 YW50 Ireland; 2 Centre for Precision Engineering, Materials and Manufacturing (PEM Centre), Atlantic Technological University, ATU Sligo, Ash Lane, Co. Sligo F91 YW50 Ireland; 3 Materials Research Institute, Technological University of the Shannon: Midlands Midwest, Athlone, N37HD68 Ireland

**Keywords:** Mechanical properties, Dissolution profile, PAT, Real-time monitoring, Drug delivery systems, Machine learning, Polymeric drug delivery

## Abstract

In the production of polymeric drug delivery devices, dissolution profile and mechanical properties of the drug loaded polymeric matrix are considered important Critical Quality Attributes (CQA) for quality assurance. However, currently the industry relies on offline testing methods which are destructive, slow, labour intensive, and costly. In this work, a real-time method for predicting these CQAs in a Hot Melt Extrusion (HME) process is explored using in-line NIR and temperature sensors together with Machine Learning (ML) algorithms. The mechanical and drug dissolution properties were found to vary significantly with changes in processing conditions, highlighting that real-time methods to accurately predict product properties are highly desirable for process monitoring and optimisation. Nonlinear ML methods including Random Forest (RF), K-Nearest Neighbours (KNN) and Recursive Feature Elimination with RF (RFE-RF) outperformed commonly used linear machine learning methods. For the prediction of tensile strength RFE-RF and KNN achieved *R* ^2^ values 98% and 99%, respectively. For the prediction of drug dissolution, two time points were considered with drug release at t = 6 h as a measure of the extent of burst release, and t = 96 h as a measure of sustained release. KNN and RFE-RF achieved *R* ^2^ values of 97% and 96%, respectively in predicting the drug release at t = 96 h. This work for the first time reports the prediction of drug dissolution and mechanical properties of drug loaded polymer product from in-line data collected during the HME process.

**Supplementary Information:**

The online version contains supplementary material available at 10.1186/s42252-024-00063-5.

## Introduction

 In vitro dissolution testing is considered one of the key analytical procedures in the pharmaceutical industry for quality assurance and is extensively used to predict the in vivo drug release. In the literature many factors have been found to influence the dissolution profile in polymeric drug delivery systems including, particle size distribution, API (Active Pharmaceutical Ingredient) content, API moisture content, polymer content, and processing conditions [1–3]. At the product development stage, in vitro dissolution testing is used to monitor physical changes in the API, to assess product variability from batch-to-batch, and help to optimise the formulation to achieve the desired release profile [4]. As such, dissolution profile is considered an important Critical Quality Attribute (CQA) throughput the development life-cycle of product release [5]. Similarly, mechanical properties are also considered important CQA of pharmaceutical materials. For example, important mechanical characteristics that affect the tabletting process include tensile strength, elasticity, hardness, and fracture toughness of the drug loaded polymeric matrix [6]. For application in drug eluting implants, mechanical properties are important to investigate the suitability for downstream processing (such as 3D printing) and to assess the suitability of a given material for desired application [7].

To analyse mechanical properties and dissolution profile, currently the pharmaceutical industry uses a well-established batch-based offline lab testing approach for quality control. Such methods are destructive, labour intensive, and time and cost consuming. In particular, dissolution testing of slow and sustained release formulations is prolonged, and a large amount of solvent is also involved. As an alternative to time and cost consuming lab-based testing, the potential for real-time monitoring and prediction of dissolution profile and mechanical properties using PAT (Process Analytical Technology) can be explored. In recent years, machine learning methods have shown huge potential to explore many different aspects of pharmaceutics. Machine learning methods can potentially be applied during all stages of drug discovery and development including clinical trials [8–10].

Hot Melt Extrusion (HME) is considered a well-established pharmaceutical manufacturing process with ability to integrate PAT tools for process monitoring. In-process Raman, NIR, and UV-Vis have been widely explored in the literature for real-time process monitoring in HME [11, 12]. In-line sensors coupled with machine learning methods have been extensively used for real-time drug quantification [13–19], and for prediction of the solid-state of the polymer-drug product [20–23] drug uniformity [24]; degradation of drug [25]; and degradation of polymer [26–29].

For prediction of the dissolution profile, offline NIR spectroscopic analysis of the product coupled with machine learning methods has been used in several studies. NIR spectra from intact tablets were used with linear regression methods including Partial Least Squares (PLS) and Principal Component Regression (PCR) for the prediction of tablet disintegration and drug dissolution profile [30–33]. These studies showed good potential of PLS and PCR to predict drug dissolution profile from off-line NIR spectra. More recent works, indicate the ability of Artificial Neural Networks (ANNs) to predict the drug dissolution profile in a tabletting process using a combination of process data and spectral data collected from the produced tablets using off-line NIR and Raman data [4, 34, 35]. Performance of an ANN model was compared with PLS, SVM (Support Vector Machine), and ERTs (Ensemble Regression Trees), with the ANN model giving the best predictive performance [4, 34, 35]. Galata et al., [1], used Convolutional Neural Networks (CNN) to process Raman chemical images to predict release profile. Yang et al. [36], compared the predicted performance of DNN (Deep Neural Networks) with commonly used regression methods including RF, SVR, KNN and PLS and MLR (Multiple Linear Regression) for the prediction of dissolution profile of sustained and released and oral fast disintegrating films. They developed machine learning models using formulation and processing data and reported to achieve excellent accuracy with DNN along with reasonable accuracy achieved by SVR and KNN. Bannigan et al. [37], compared the ability of different machine learning methods to predict fractional drug release from polymeric long acting injectables from formulation and process settings data. Out of all methods Light Gradient Boosting Machine (LGBM) showed best performance to predict release profile. All these works highlight the ability of machine learning methods to predict dissolution profile from off-line spectral data of tablet products, together with process settings and formulation data. Pawar et al. [38] for the first time used NIR at-line for the prediction of drug release profile in a continuous direct compaction process for acetaminophen tablet preparation. Acetaminophen concentration, blender speed, feed frame speed and compaction force were used as variables to conduct the experiments. NIR spectra collected at-line from all experiments were processed using multivariate linear regression to predict the dissolution profile at specified time points. The model achieved reasonable accuracy to predict the release profile of individual tablets and yielded similarity factor *f*_*2*~_72%. This work highlights the ability of using NIR as PAT tool during processing and achieving good predictive accuracy for dissolution profile. However, in-line application is preferable to at-line due to more rapid feedback, continuous monitoring, and elimination of the need for human intervention in taking or preparing samples. However, it adds significant complexity as in-process NIR spectra are subject to additional sample variations in temperature, pressure, viscosity etc.

In the case of mechanical properties, in-line NIR together with other process data has previously been reported to be effective for predicting the mechanical properties of extruded polymers using different Machine Learning techniques [39–42]. However, to date, no work has been reported on the prediction of mechanical properties or dissolution profile of a drug-loaded polymeric matrix using in-process sensor data. Real-time release testing with the help of a PAT tool is more desirable for continuous manufacturing environment [43].

In this work Polylactic acid (PLA) is used as the drug carrier. PLA has been used in a range of different applications including implants for cardiovascular, orthopaedic, and dental applications. PLA microspheres and PLA nanoparticles are used for drug delivery devices, particularly for delivery of anticancer drugs. Other applications of PLA include biodegradable needles, screws, plates, sutures etc., [44–46]. Aspirin (Acetylsalicylic acid) was used as a model drug with PLA. Aspirin (INN,ASA), which is an NSAID (Non-Steroidal Anti-Inflammatory Drug) [47], is one of the most widely used drugs in the world. As an alternative to oral delivery of INN,ASA which has low bioavailability, INN,ASA has been used with polymeric implants to develop local implantable drug delivery devices for applications in osteogenesis [48–50] to prevent thrombosis [51], to prevent cardiovascular events [52, 53], and for reducing implant associated infections etc. [54].

**Fig. 1:**
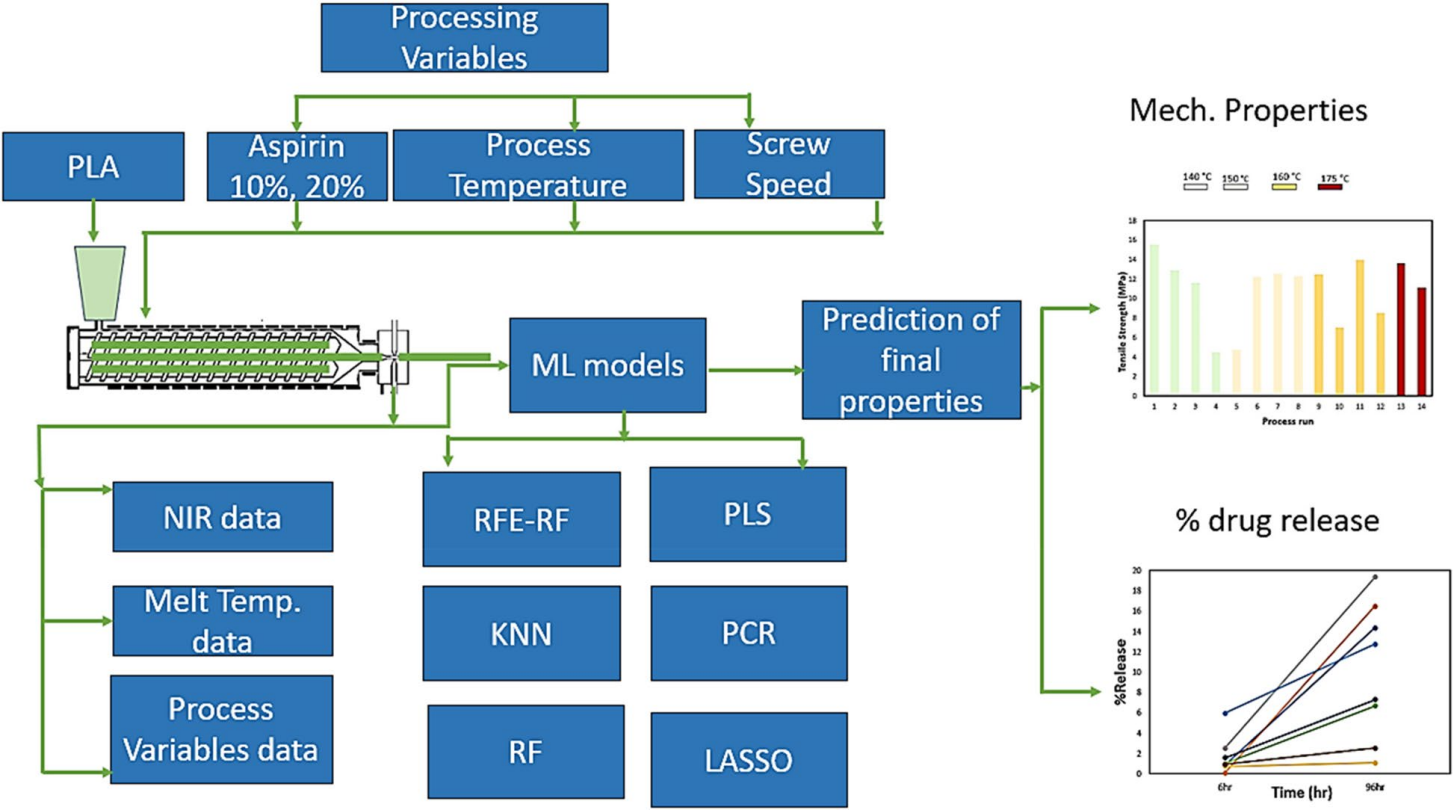
Schematic Representation of the methodology

A twin-screw extruder is used to process PLA-INN,ASA at a range of different processing conditions, with the aim of developing a soft sensor model using in-line NIR, melt temperature sensor data and process setting data for the prediction of drug dissolution profile, tensile strength, and elongation at break. Based on previous literature for PLA-INN,ASA systems, two time points were selected for drug dissolution prediction to capture burst release (6 h) and sustained release (96 h) respectively. A high burst release can be desirable in some applications (e.g. wound healing) but more generally a controlled sustained release is preferred and hence monitoring and control process of these to achieve the desired kinetics is desired. The results of various Machine Learning methods for the prediction of dissolution profile and mechanical properties were compared using a robust Monte-Carlo Cross Validation (MC-CV) approach. The non-linear machine learning methods showed excellent accuracy in prediction of drug dissolution and the mechanical properties of the drug loaded PLA matrix. Figure 1 shows the schematic representation of this work. To the best of the authors’ knowledge, this is the first report in the literature indicating that the CQAs of dissolution profile and mechanical properties of a drug loaded polymer carrier can be predicted from in-process data using machine learning methods.

## Materials

**Fig. 2:**
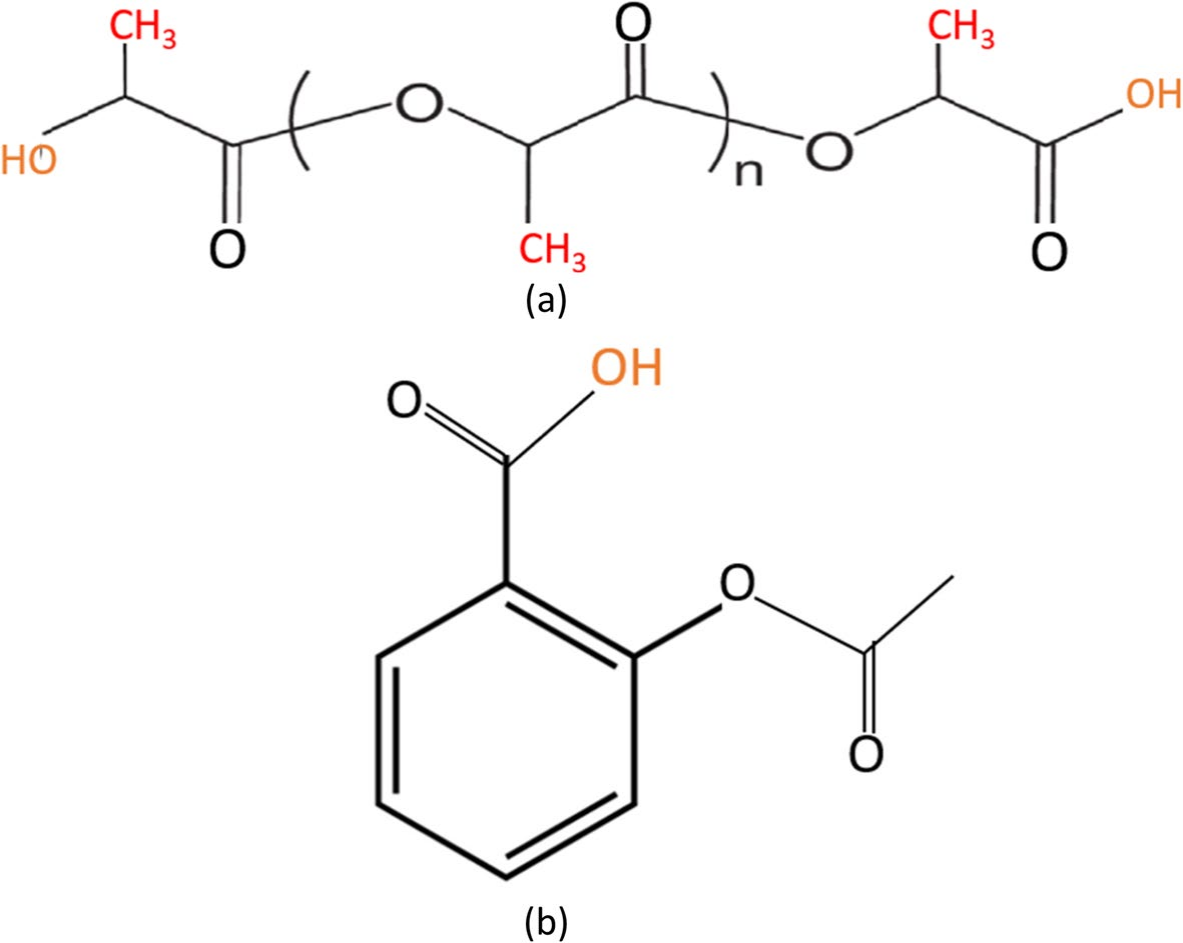
Chemical structure of **a** PLA, **b** INN,ASA

Packaging grade PLA (2003D) was purchased from NatureWorks, LLC. INN,ASA (Acetylsalicylic acid > 99%) was purchased from Fisher scientific, UK. Figure 2a & b show the structure of PLA and INN,ASA.

## Experimental details and in-line sensors

PLA was dried at 65 °C for two hours prior to experiments. A mortar and pestle was used to thoroughly mix PLA with INN,ASA (Aspirin) before extrusion. A 16 mm co-rotating Prism twin screw extruder with length to diameter ratio of 25:1 was used to process PLA-INN,ASA (PLA-ASP) mixture. The extruder barrel was divided into four sections operated at different temperatures. An adapter and slit die were attached (termed zone 5 and 6, respectively) to the end of the extruder to capture in-process data using NIR and temperature sensors.

Pure ASA has a melting point between 136 and 140 °C, and DSC data for ASA shows a broad endothermic peak between 135 and 210 °C, which relates to the thermal decomposition of ASA over this range [55]. PLA melting point is above 155 °C and thermal degradation of PLA starts above 200 °C [56]. In the literature ASA with polymeric carriers has been processed using HME and FDM printing process between 140 and 180 °C without thermal decomposition [56–58]. This is due to the fact that when drugs are processed with polymers which have relatively higher melting point, they can help to reduce the thermal decomposition of the drugs. During the HME process, the drug is dispersed within the polymer at molecular level and polymer can act as barrier to protect drug from thermal degradation. Furthermore, drug can interact with the polymer through hydrogen bonding, by forming weak intermolecular forces (van der waals forces) or through hydrophobic interactions. These interactions help to reduce the molecular mobility of drug which can help to reduce thermal degradation and stabilise thermolabile drugs [58–60]. Moreover, shear stresses, residence time and moisture content in the polymer also play a crucial role in the thermal decomposition of polymer-drug system during melt extrusion [61].

A Design of Experiment (DoE) methodology was used to design the experiments. A multilevel factorial design was used to study the effect of screw speed, temperature, and INN,ASA concentration on the final properties of PLA-INN,ASA matrix. Three factors including temperature, screw speed, and drug concentration were used. Based on the literature, three levels for temperature (140 °C, 150 °C, and 160 °C), two levels for screw speed (6 Hz and 8 Hz) and two levels for drug concentrations (10% and 20% INN,ASA) were used, giving a total of twelve process runs. Two additional runs were also carried out at 175 °C (with 20% INN,ASA only) to analyse whether at this elevated temperature mechanical properties and release profile would be significantly affected. PLA with INN,ASA could not be processed below 140 °C. It resulted in exceeding extruder torque limit and also resulted in extremely poor/inhomogeneous melting. So, in total 14 experiments were conducted. All samples were air cooled post-extrusion. For all samples, extruder torque was within the limit of the machine and no issues arose during processing. However, processing of samples at 175 °C was somewhat challenging as the product was very sticky. Table 1 lists the formulation and processing conditions for all experiments.

**Table 1:** Processing conditions for experiments

Process run	%INN,ASA (%ASP)	zone1	zone2	zone3	zone4	zone5	zone6	Screw speed
		°C	°C	°C	°C	°C	°C	Hz
one	10	95	100	115	130	140	140	8
two	10	95	100	115	130	140	140	6
three	20	95	100	115	130	140	140	6
four	20	95	100	115	130	140	140	8
five	10	95	100	115	130	150	150	8
six	10	95	100	115	130	150	150	6
seven	20	95	100	115	130	150	150	6
eight	20	95	100	115	130	150	150	8
nine	10	95	100	115	130	160	160	8
ten	10	95	100	115	130	160	160	6
eleven	20	95	100	115	130	160	160	6
twelve	20	95	100	115	130	160	160	8
thirteen	20	95	100	115	130	175	175	8
fourteen	20	95	100	115	130	175	175	6

In our previous work on the prediction of yield stress of pure PLA processed via HME, it was determined that NIR along with conventional process sensors provides significantly better accuracy to predict the yield stress compared to using NIR or pressure and temperature sensors alone [39]. Hence, in this work, the slit die at the end of the extruder was also equipped with in-line NIR, and melt temperature sensors. Two temperature sensors termed T1 and T2 were inserted in the die (on the top and bottom) to record the temperature profile. Each transducer was embedded with type K thermocouples. The NIR spectroscopy system consisted of two fibre-optic probes measuring NIR spectra in the wavenumber range of 4000–7500 cm^-1^ with a resolution of 1 cm^-1^. NIR spectra were collected using Interspectrum software. The temperature data was recorded at a sample frequency of 5 Hz while NIR spectra were acquired every 5 s. The NIR spectroscopy system and temperature sensors were supplied by FOS Messtechnik GmbH.

## Characterisation

### Mechanical testing

Three samples were taken in each process run from different portions of the extruded film and characterised for mechanical testing. Tensile strength and elongation at break for each sample were measured using Zwick Roell Z0.5 tensile tester with a load cell of 500 N. Tests were carried out at a speed of 5 mm/min, with a measurement accuracy of ± 1%.

### Dissolution testing

DISTEK 2100 basket dissolution apparatus (Distek, Inc. North Brunswick, NJ, USA) was used to study the dissolution profile of the PLA-ASP samples. Pre-weighed PLA-ASP samples were placed inside the tank. The weight of the PLA samples with 10% and 20% INN,ASA was approximately 4gm for all samples. Based on the solubility data and the actual weight of INN,ASA in the sample, dissolution baths were filled with 900 ml Phosphate Buffer Solution (PBS) with pH 7.4. The apparatus was set at 37 °C and 50 rpm. After taking a 2 mL sample, tanks were replenished with same amount of PBS solution to keep the volume of the PBS constant during the process. Samples were analysed using a UV-vis spectrophotometer (Shimadzu Europa GmbH, Duisburg, Germany). Absorbance of all samples were recorded at wavelength 298 nm [47, 58]. A standard calibration curve for INN,ASA was constructed and standard procedure was used to calculate the dissolution profile of all samples at specified time points [62].

### Differential scanning calorimeter (DSC)

DSC Perkin Elmer 4000 (Perkin Elmer Washington, MA, USA) was used to study the thermal behaviour of PLA-ASP samples. Three cycles were used, in the first cycle the sample was heated from 30 to 250 °C using 10 °C/min heating rate. Then, in the cooling cycle, the sample was cooled down from 250 –30 °C using a 10 °C/min cooling rate. This was followed by a second heating cycle at the same conditions as the initial heating cycle. Sample measurements were recorded in a nitrogen atmosphere. %Crystallinity (X_C_) of all the samples was calculated using Eq. (1).

1 $$\:\text{X}\text{c}=\:\frac{{\Delta\:}{\text{H}}_{\text{m}}}{{\Delta\:}\text{H}{^\circ\:}_{\text{m}}(1-\text{w}\text{f})}\text{*}100\:\:$$

Whereas,

$\:{\Delta\:}{\text{H}}_{\text{m}}$= measured melting enthalpy

$\:{\Delta\:}\text{H}{^\circ\:}_{\text{m}}$= enthalpy of 100% crystalline PLA (93.7 j/g)

$\:\text{w}\text{f}$ = weight fraction of drug in PLA

## Selection of input data

To develop the models for the prediction of mechanical properties and dissolution profile, NIR readings for all samples were included. Approximately forty-four NIR spectra were collected for each process run. In total 601 wavenumbers from spectral region 6100–6700 cm^-1^ were included in the model. Melt temperatures readings from the thermocouples in the last section of extruder (die) were also included as input features. As little variation was observed in these melt temperature values during the process, only the mean melt temperature value for each thermocouple was included for each process run. Process setting data including extruder temperature settings and screw speed were also included as input features. The final size of the data set was *n* = 628, *p* = 610.

**Fig. 3:**
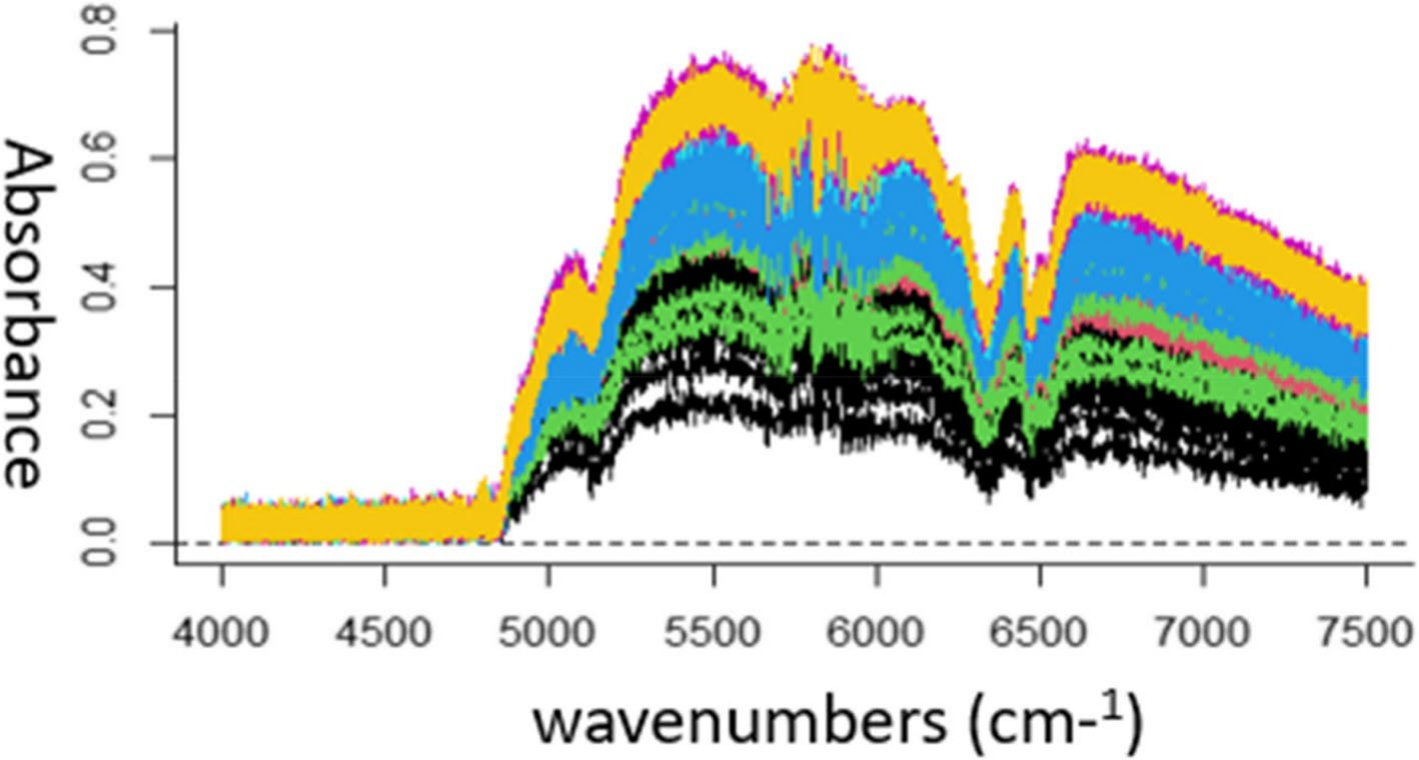
NIR spectra of all samples

Figure 3 shows the NIR spectra for all process runs. To develop the models, only NIR spectra region 6100–6700 cm^-1^ was selected. For PLA, this spectral region is known to be the first overtone region of C-H stretching, associated with C-H, O-H, C = O bond activities, and provides information about molecular chains orientation, degradation and crystallinity [63]. These PLA properties affect the mechanical properties and dissolution profile of the product [64]. In our previous work for the prediction of yield stress of pure PLA from in-line NIR data, the same NIR spectral region was used and resulted good predictive accuracy [39, 65]. For INN,ASA, the spectral region 6100–6700 cm^-1^ is linked with first overtone of C-H stretching in the benzene ring [66, 67]. Hence, this spectral region is anticipated to be highly relevant for the quantification of mechanical and dissolution properties of PLA-INN,ASA matrix. It is highly beneficial to select only relevant spectral region/s to develop model instead of using the full spectrum which is likely to contain non-informative regions. Including irrelevant input features is known to increase model complexity and increases the risk of model overfitting. In the literature, different strategies for automating the identification of relevant spectral regions have been proposed, of which iterative PLS (iPLS) approaches are the most common. Here we compare an automated backwards iPLS (BiPLS) method against our selection of the most relevant region based on chemical knowledge.

All spectra were pre-processed using baseline corrections and Multiplicative Scatter Correction (MSC) to remove baseline shifts and undesired scatter effects [68]. Machine learning regression algorithms for prediction of mechanical properties and drug release profile included: PLS, Random Forest (RF), *K*-Nearest Neighbours (KNN), and feature selection methods including Recursive Feature Elimination (RFE), and Least Absolute Shrinkage and Selector Operator (LASSO) were used. For the prediction of the mechanical properties, the mean value of the triplicate tests for tensile strength and elongation at break for each process run were used as target variables. As INN,ASA loaded PLA films exhibited burst release in first 6 h followed by sustained release up to 96 h, %drug released at t = 6 h and t = 96 h were used as target variables for the prediction of dissolution profile.

### Training and validation of models

A Monte-Carlo Cross Validation (MC-CV) approach was used for robust validation of model performance. MC-CV is shown to outperform other cross-validation approaches such as Leave One Out (LOOCV) or *k*-fold CV in terms of the probability of selecting the correct number of components to include in the model and in terms of estimating the true prediction error. While LOOCV is widely used with small data sets, it has been shown to tend towards selecting too many components to include in the model, resulting in overfitting and over estimating the true prediction accuracy. To have a stable estimation of model performance, a good balance between training and test set is required [69, 70]. In MC-CV a high number of train-test iterations is used. At each iteration, a different random split into training and test sets is applied. The test set is selected to investigate the predictive accuracy of the model and should generally be between 30% and 50% of the data [69]. This procedure is repeated several times, usually 100 or more [70]. As a higher number of iterations in MC-CV can be used compared to *K*-fold cross validation, MC-CV provides better estimates of model performance for differently selected unseen test sets. However, due to the high numbers of iterations, MC-CV is computationally expensive compared to *K*-fold cross validation [69, 71]. Here, the MC-CV approach was used to select the spectral region that would yield best predictive accuracy, and also to investigate the robustness of the different Machine Learning regression methods. In this work, 100 iterations of training and testing was carried out for cross-validation. At each iteration, 30% of the data was randomly selected as the test set to assess the predictive performance of the model trained on the other 70%. This gives 100 different values of RMSE (Root Mean Squared Error) and *R* ^2^ on different test sets. The standard deviations of the RMSE and *R* ^2^ give a measure of the robustness of the models. A high standard deviation indicates a high sensitivity to how the data is split for training and testing and hence is an indicator that the model is not robust.

As endorsed by FDA guidelines, the reliability of machine learning predictive models should be assessed considering accuracy (closeness of agreement between actual and predictive values, i.e., represented by predictive errors), linearity (predicted vs. measured values), and precision (standard deviation of predictions) [72]. The mean RMSE (indicator of accuracy); the standard deviation of the RMSE (indicator of precision); the mean *R* ^2^ (Correlation coefficient between actual and predicted values as an indicator of linearity); and standard deviation of *R* ^2^ were computed to assess the performance of each predictive model. A Normalised Root Mean Squared Error (NRMSE) was also calculated by dividing the RMSE by the range of the actual values of target variables, this gives a good indication of the model sensitivity relative to the amount of variation exhibited in both mechanical properties and the drug dissolution. The soft sensor was modelled using R (version 4.2.2) software as a back end and RStudio (version 2022.12.+353) software. Modelling script is provided in the Supplementary data.

## Results and discussion

### DSC analysis

Table 2 lists melting temperatures (T_m_), onset melting temperatures (T_onset_) and melt enthalpy of all the samples. Melting temperature is the temperature at which the sample undergoes significant phase transition from solid to liquid, while the onset melt temperature of a sample is defined as the temperature at which the sample chains begins to melt. For pure INN,ASA the melt peak was identified at 144.35 °C with onset melt temperature 139.37 °C. For pure PLA processed at the lower screw speed, melting started at a slightly higher temperature of 154.42 °C than for pure PLA processed at higher screw speed which had a melt temperature peak 150.89 °C. This may be because screw speed is crucial in determining the residence time and ultimately exposure of polymer matrix to heat and shear stresses. At lower screw speed, longer residence time, extended exposure to shear stresses can result in enhanced molecular orientation and crystallinity which can shift the melting temperature to higher value. This was supported by DSC results as PLA sample processed at lower screw speed exhibited higher crystallinity (44%) than PLA sample processed at higher screw speed (Xc = 38%).

**Table 2:** DSC results of PLA-INN,ASA samples for process runs 1-14

**Process run**	**%INN,ASA**	**Die Temperature**	**Screw speed**	**Melt Temp (T**_m_**)**	**Onset Temperature (T**_onset_**)**	**Crystallinity (%Xc)**
		°C	Hz	°C	°C	
Pure INN,ASA	**-**	-	-	144.35	139.37	
PLA		160	6	154.42	143.44	44
PLA		160	8	150.89	143.52	38
One	10	140	8	147.09	143.21	46.79
two	10	140	6	154.42	143.54	42.68
three	20	140	6	141.09	132.14	30.58
four	20	140	8	139.3	125.19	32.70
five	10	150	8	144.75	131.72	28.88
six	10	150	6	147.91	130.29	36.74
seven	20	150	6	147.75	134.08	28.37
eight	20	150	8	143.74	137.25	34.47
nine	10	160	8	146.27	135.91	39.53
ten	10	160	6	148.96	141.13	36.62
eleven	20	160	6	146.25	137.57	37.77
twelve	20	160	8	140.41	127.65	33.16
thirteen	20	175	8	139.44	125.67	30.28
fourteen	20	175	6	139.05	123.11	37.48

Similarly, for all PLA-INN,ASA samples, process runs at high temperature with high screw speed and high INN,ASA loadings (P12, P13, P14) exhibited the lowest melt temperature. For all PLA-INN,ASA samples, the melting temperature was found to be between 139 and 147 °C, this shows that the presence of INN,ASA in the PLA reduced the melt temperature. It can also be seen from the Table that for all processing conditions, samples with 20% INN,ASA loading showed slightly lower melting temperature than samples with 10% INN,ASA loading. This shows that a higher concentration of INN,ASA reduced the melting temperature of the sample. Moreover, for pure PLA the melting peak was detected around 150–154 °C while for all PLA-ASP samples, the melting temperature was found to be between 139 and 147 °C. The decrease in melting point suggests interaction between PLA-INN,ASA. The melting point is also influenced by molecular weight and crystallinity of the PLA. It was observed that addition of INN,ASA reduced the crystallinity of PLA. Figure 10 (provided in Supplementary data) shows a single glass transition peak for all PLA-INN,ASA samples. This suggests that there is a miscibility between the PLA and INN,ASA. If PLA-INN,ASA has not interacted during process, two separate glass transition peaks would be observed. To summarise, the DSC results show interaction between PLA-INN,ASA samples, and suggest that different processing conditions affect the molecular orientation and crystallinity.

**Fig. 4:**
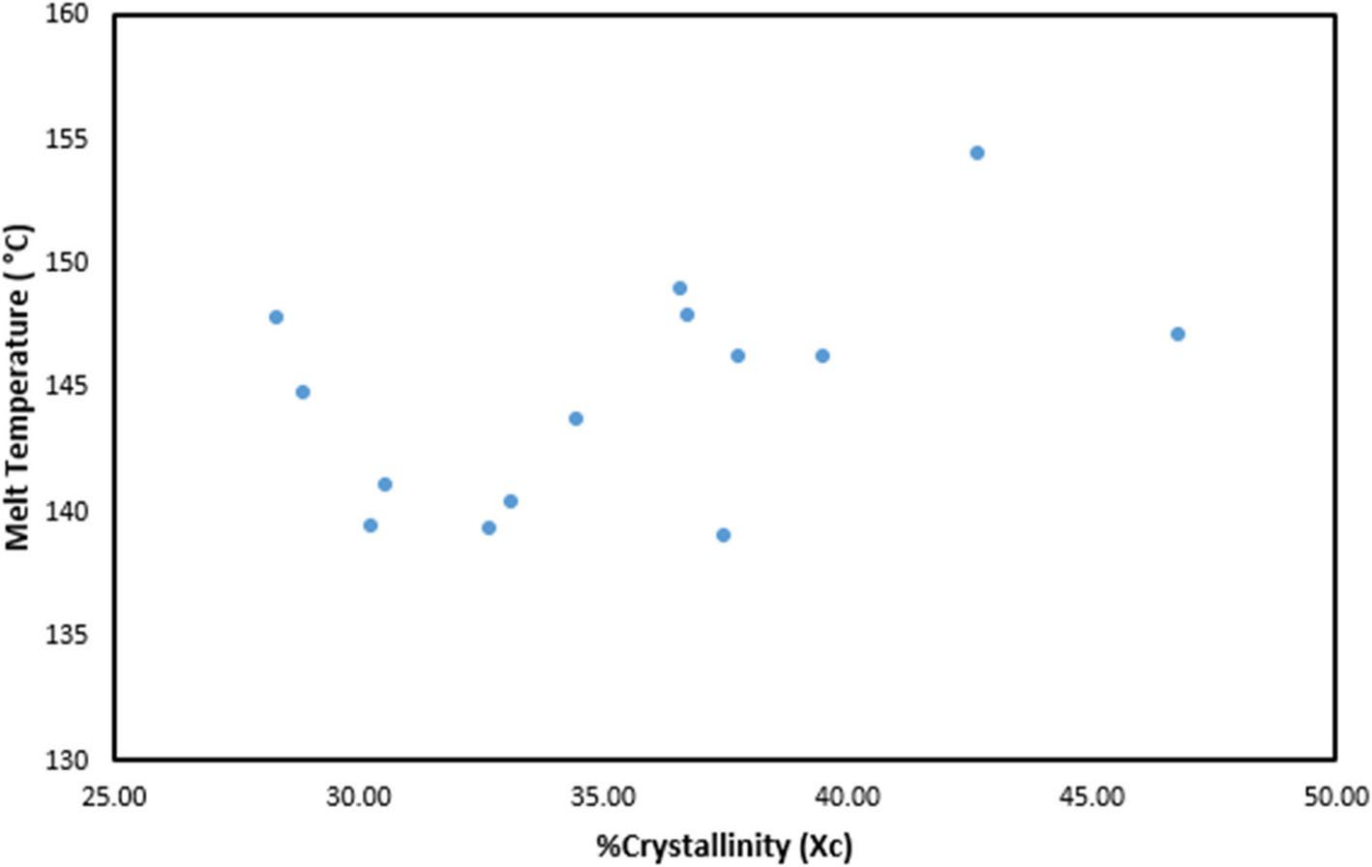
PLA-INN,ASA melt temperature vs. %crystallinity

Moreover Fig. 4 shows the relationship between crystallinity and melting temperature for all samples. It can be seen that samples with %crystallinity higher than 35% showed a higher melting temperature than samples with %crystallinity less than 35%. However, some outliers, for example process run 14, 7 and 5, showed opposite trend. A strong positive relation between %crystallinity and melting temperature was not be observed.

### Effect of process settings on mechanical properties

**Fig. 5:**
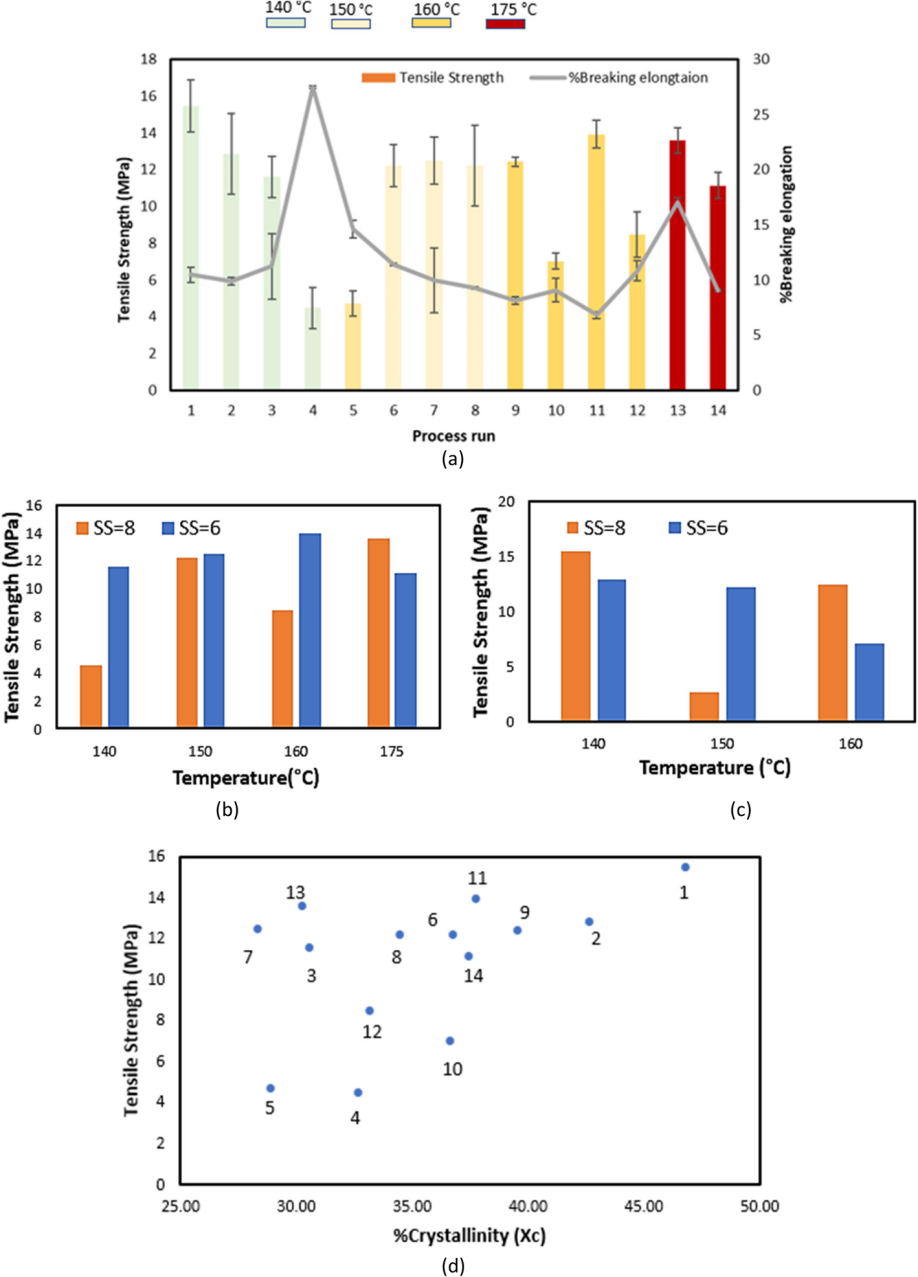
Effect of processing variables (**a**) temperature on tensile strength, and **b** screw speed, on tensile strength with 20% ASP; **c** effect of screw speed on tensile strength with 10% INN,ASA, **d** crystallinity vs. tensile strength

Despite the many benefits of PLA in drug delivery applications, processing of PLA using the HME process is challenging as PLA tends to degrade in the presence of high temperature and mechanical stresses. Moreover, PLA feedstock is also very sensitive to environmental conditions (humidity), and presence of moisture in the feedstock can lead to hydrolytic degradation during processing which ultimately affects the final properties of product. For process optimisation and for consistent product quality, it is essential to understand the effect of process variables on the final properties of product. However, for the HME process understanding the effect of processing conditions on the final product properties is complex [61]. In this work, with changing processing conditions, significant variation in tensile strength (standard deviation of 3.40 MPa) was observed across different process runs (Fig. 5a). While a correlation between process temperature and tensile strength might have been expected (due to degradation at high temperatures), this was not observed. During the HME process, the effect of process variables is coupled, e.g., the melt temperature is affected by both screw speed and temperature settings. These parameters affect the thermal properties such as onset and melting temperature (as listed in Table 2) of polymer-drug matrix. These parameters ultimately affect mixing, residence time and final properties e.g., mechanical properties, and it is not possible to determine optimum process settings from single factor analysis. For example, for 10% ASP, samples processed at lowest processing temperature with high screw speed (140 °C, 8 Hz) showed highest strength among all samples. However, samples processed at 160 °C showed better strength than samples processed at 150 °C (see Fig. 5b & c).

Additionally, the %Crystallinity of all the samples was analysed to investigate the effect of crystallinity on the tensile strength. %crystallinity of all the samples was calculated using Eq. (1). Significant differences in %crystallinity were observed for samples processed at different processing conditions. The lowest %crystallinity value observed was 28% and the highest %crystallinity value was 46%. A standard deviation of 5.1% was found between all samples. Crystallinity is considered an important property of drug loaded polymeric products that directly influences the mechanical properties of the product. Generally highly crystalline samples will have high mechanical strength [73]. In Fig. 5d, a positive correlation between crystallinity and tensile strength can be observed i.e., among all samples, the highest crystallinity was recorded for process run 11, which exhibited the second highest tensile strength among all samples.  Process run five which exhibited the lowest tensile strength among all samples also exhibited the lowest %crystallinity. However, while there is a positive correlation between crystallinity and tensile strength, overall, the correlation is not very strong and outliers were observed for some process runs.

Based on these results, it can be seen that the effect of individual process variables on the mechanical properties and correlation between product attributes is not consistent, and these does not provide enough information to be certain about materials behaviour and product properties under different processing conditions. In-process sensors such as NIR on the other hand, can provide information about the effect of process variables on the product quality in real-time and can help to understand materials behaviour during processing in a better way.

### Effect of process settings on the dissolution profile

Polymeric drug delivery systems are mostly used to achieve a controlled release rate for targeted drug delivery. The benefit of using a controlled release formulation is that it achieves the same therapeutic effect with less frequent drug dosing than conventional drug delivery systems [68, 69, 74, 75]. Most of the controlled release formulations release drug through two different modes: burst release and sustained release. In sustained release, the drug/API is released over a prolonged period of time e.g., days/weeks/months etc. However, in burst release, an appreciable amount of drug/API is released immediately when drug comes in contact with the release media [76]. In the literature most of the controlled released formulations have shown some degree of initial burst release [70–72, 76–78]. However, initial burst release is generally considered undesirable as it reduces the lifetime of the drug delivery devices (as sustained drug release is reduced), can cause toxicity, and means the patient would require frequent drug doses [76]. On the other hand, burst release in some applications is considered beneficial e.g., wound treatment, targeted burst release [76]. However, it is also considered challenging to predict burst release or the actual amount of drug that would be released in the initial burst while designing the drug delivery devices [76].

**Fig. 6:**
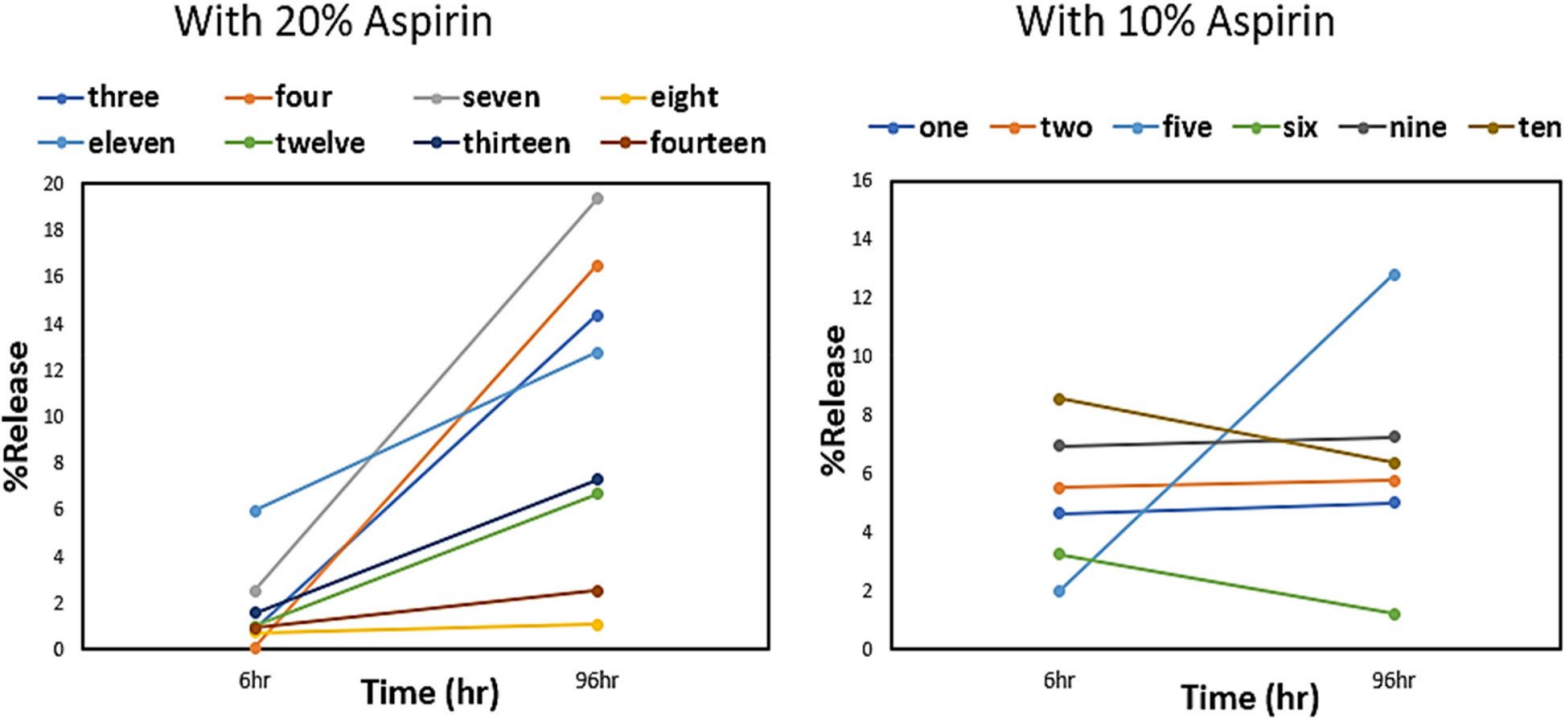
Dissolution profile of PLA-INN,ASA samples with time for process runs 1-14 (**a**) with 10% INN,ASA, **b** with 20% INN,ASA

It is essential to select optimal processing conditions for designing a polymer drug delivery system for controlled release. To select optimal processing conditions, it is necessary to understand the relationship between processing and formulation parameters and drug release profile. In this work, the effect of screw speed, temperature, and concentration of drug on the %drug released was studied at two-time points t = 6 h, and t = 96 h. Figure 5 shows %drug release of all the samples. Up to time t = 6 h the percentage of drug released was considered as a metric for comparing degree of initial burst release. The percentage of drug released from t = 6 h up to t = 96 h was considered a suitable metric to compare the sustained release [58]. This is based on similar release profiles for INN,ASA loaded PLA samples prepared using HME process has been reported by Venkatesh at el*.* [58]. Their samples showed a burst release in the initial 8 h followed by a slow release until 72 h and after 72 h samples achieved a sustained release profile [58]. In this work all process runs conducted at different processing conditions vary from each other in terms of %drug release. Drug concentration and mixing within the polymer matrix play important role in describing initial burst release and drug release. As can be seen in Fig. 6, after the initial 6 h, for samples with 10% INN,ASA loading, approximately 2–8% drug was released. For samples with 20% INN,ASA loading, 0.7-6% drug was released after 6 h. Samples with 20% INN,ASA processed at temperatures 150 °C and 160 °C (i.e. higher than the melting point of the drug), resulted in greater %drug release, and samples processed at around the melting point of INN,ASA (140 °C ) resulted in very low %drug release after 6 h. After 96 h all samples showed a significant increase in %drug release compared to the release at 6 h, and significant variation in %drug release among samples processed at different processing conditions was observed.

**Fig. 7:**
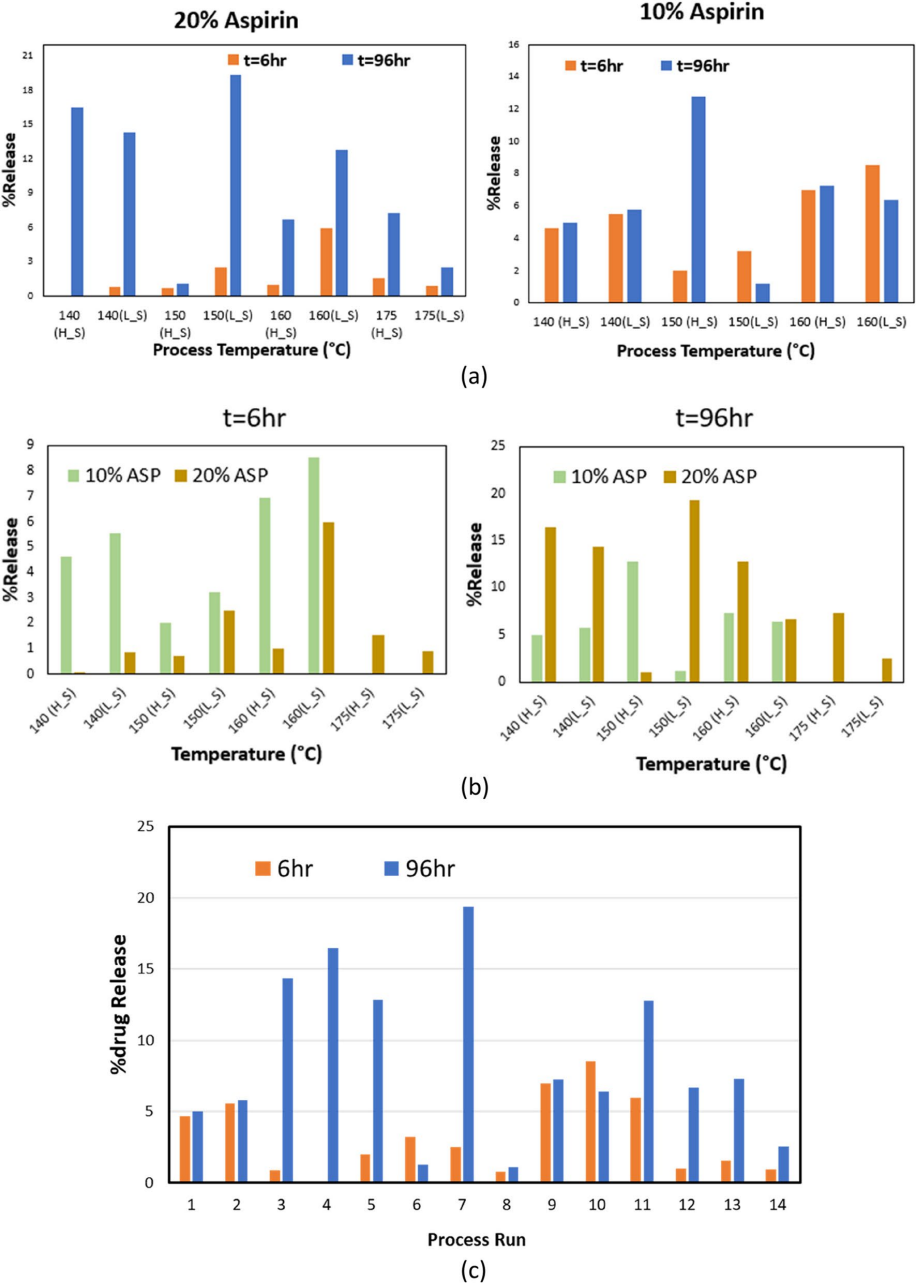
Effect of process variables on PLA-INN,ASA samples for process runs 1-14 (**a**), temperature and screw speed, (**b**) % of drug released. (L_S) indicatse process runs at lower screw speed (6Hz), and (H_S) indicates process runs at high screw speed. **c** %release for process run 1-14, processed at different conditions

Varying processing conditions i.e., temperature and screw speed can be seen to have a significant effect on the drug release profile. Figure 7a shows the effect of process temperature and screw speed on the release profile for samples with 10% and 20% INN,ASA. Samples with 10% INN,ASA processed at different conditions showed significant variation in %drug release. At t = 96 h, for samples with 10% INN,ASA loading, a standard deviation of 4.01% drug release was observed in the samples produced at processing temperatures from 140 °C to 160 °C. The highest %drug release of 12.81% was observed at 150 °C, and lowest %drug release of 5% was observed at 140 °C. For samples with 20% INN,ASA, at t = 96 h, with the increase of temperature from 140 °C to 175 °C a standard deviation of 6.37% was observed. Highest %drug release of 16.46% was achieved at 140 °C, and lowest %drug release of 1.07% was observed at 150 °C.

The effect of screw speed and temperature are coupled and also depend on the drug content. Figure 7b shows the effect of drug concentration on the drug release profile. In some cases, lower screw speed resulted in greater %drug release, but an opposite trend was observed for other cases. For example, for both formulations greater %drug release was observed at t = 6 h in samples processed at the lower screw speed for all processing temperatures. However, at t = 96 h, the higher screw speed resulted in greater %drug release than the lower screw speed for all of the 10% INN,ASA samples, while for the 20% samples temperature was also a factor. At t = 6 h, samples with 10% INN,ASA loading showed greater %release than sample with 20% INN,ASA loading at all processing conditions. However, at t = 96 h samples with 20% INN,ASA loading showed greater %release than samples with 10% INN,ASA loadings for most of processing conditions with the exception of samples processed at 150 °C at high screw speed (Fig. 7b). Figure 7c shows the variation between percentage release for all process runs processed at different conditions for time t = 6 h and t = 96 h. It can be seen that different processing conditions significantly affect the percentage release profile. However, it is difficult to understand the effect of processing conditions using single factor analysis.

In summary, there are no clear relationships for selection of optimal processing conditions to achieve a specific profile of burst and sustained drug release. This is because for the HME process, specifically in the case of biodegradable polymer drug delivery systems, processing conditions including screw speed, processing temperature and also the melt temperature affect the thermal properties (glass transition temperature (Tg), melting temperature), degree of crystallinity and mixing of polymer and drug [79]. These parameters in turn affect drug degradation, drug solubility and ultimately drug release behaviour [58, 73, 79]. In terms of the effect of formulation (different loading of INN,ASA), INN,ASA is known to have plasticising effect, which is the decrease in glass transition temperature during processing. Hence, different drug concentration will result in different release behaviour [80]. Thus, for HME process effect of process and formulation parameters is coupled and it is challenging to investigate their effect individually and find a clear correlation.

### Prediction of mechanical properties from in-process data

 Machine learning methods were used to predict elongation at break and tensile strength from the in-process data described in Sect. 5. Elongation at break, also known as fracture strain, is an indication of the elasticity of the material. Tensile strength indicates the highest stress a material can endure before fracturing permanently. For drug-eluting implants tensile strength and elongation at break are considered important CQA to investigate the suitability for downstream processing (such as 3D printing) and to assess the suitability of a given material for desired application [7]. Tensile strength and elongation at break also affect the tabletting process and measured for quality assurance [6]. Input features of models developed for the prediction of tensile strength and elongation at break included NIR spectral region (6100–6700 cm^−1^), mean melt temperature and process settings data including screw speed and process temperatures. Machine learning models used for prediction of mechanical properties included PLS, PCR, RF, KNN, RFE-RF and LASSO. An RF model was trained using default hyperparameters (For RStudio, default number of trees (ntree) = 500, and number of variables selected at each split (mtry) = No. of input features divided by 3). For KNN, K-values used varied from 3 to 20 (with an increment of 1). RFE was used with RF as learning algorithm. For both PLS and PCR, the first three latent variables captured 98% of the variance in the data and were used as optimal number of latent variables. For LASSO, the value of penalty term λ (lambda) was varied from 0.0001 to 0.001 in increments of 0.0001.

Table 3 compares the results of all machine learning methods for the prediction of tensile strength and elongation at break for spectral region 6100–6700 cm^−1^. For tensile strength prediction, both KNN and RFE-RF achieved excellent predictive accuracy indicated by low mean RMSE, low mean NRMSE and high mean *R* ^2^ values. For KNN and RFE-RF, the standard deviation of the RMSE (0.029 MPa, and 0.086 MPa, respectively) with MC-CV is significantly lower than the standard deviation between actual tensile strength values (3.40 MPa). A low standard deviation of RMSE indicates the model’s ability to perform consistently for different unseen test sets. RFE-RF yielded a mean RMSE value of 0.54 MPa and *R* ^2^ = 97.9%. with ten features selected for inclusion in the model. KNN (K = 5) achieved excellent accuracy and yielded a mean RMSE value of 0.148 MPa with *R* ^2^ = 99.7%. RF also achieved reasonable predictive accuracy for the prediction of tensile strength. For the prediction of elongation at break, only RFE-RF and KNN performed well and achieved good predictive accuracy. REF-RF and KNN (K = 5) yielded a mean RMSE value of 1.566 AND 0.153, respectively. RF could not achieve good predictive accuracy for the prediction of elongation at break and result high mean RMSE value (2.944). Linear methods including LASSO PLS and PCR did not perform well for the prediction of tensile strength and elongation at break as indicated by high mean RMSE and low *R* ^2^ values.

**Table 3:** Monte-Carlo Cross Validation (MC-CV) for the prediction of mechanical properties (N_s= No. of features selected, N_C= No. of components/latent variables)

**Tensile Strength (MPa)**					
**Method**	**Mean RMSE (MPa)**	**SD of RMSE (MPa)**	**Mean NRMSE**	**Mean R**^2^	**SD of R**^2^
**Spectral region 6100-6700 cm**^-1^					
KNN	0.148	0.029	1.3%	0.997	0.0008
(K=5)					
RFE-RF	0.54	0.086	4.9%	0.979	0.006
(N__S_=10)					
RF (ntree=500)	1.176	0.198	10.6%	0.945	0.025
PLS	2.452	0.137	22.2%	0.372	0.076
(N__C_=3)					
PCR	3.038	0.094	27.6%	0.042	0.022
(N__C_=3)					
LASSO	3.019	0.096	27.4%	0.094	0.032
(N__S_=136)					
**Elongation at Break**					
**Method**	**Mean RMSE**	**SD of RMSE**	**Mean NRMSE**	**Mean R**^2^	**SD of R**^2^
KNN	0.153	0.0326	0.73%	0.998	0.0005
(K=5)					
RFE-RF	1.566	0.452	7.5%	0.875	0.065
(N__S_=10)					
RF (ntree=500)	2.944	0.439	14.2%	0.488	0.081
PLS	3.177	0.271	15.3%	0.396	0.03
(N__C_=3)					
PCR	3.164	0.354	17.4%	0.354	0.213
(N__C_=3)					
LASSO	3.731	0.223	18.0%	0.212	0.035
(N__S_=139)					

**Fig. 8:**
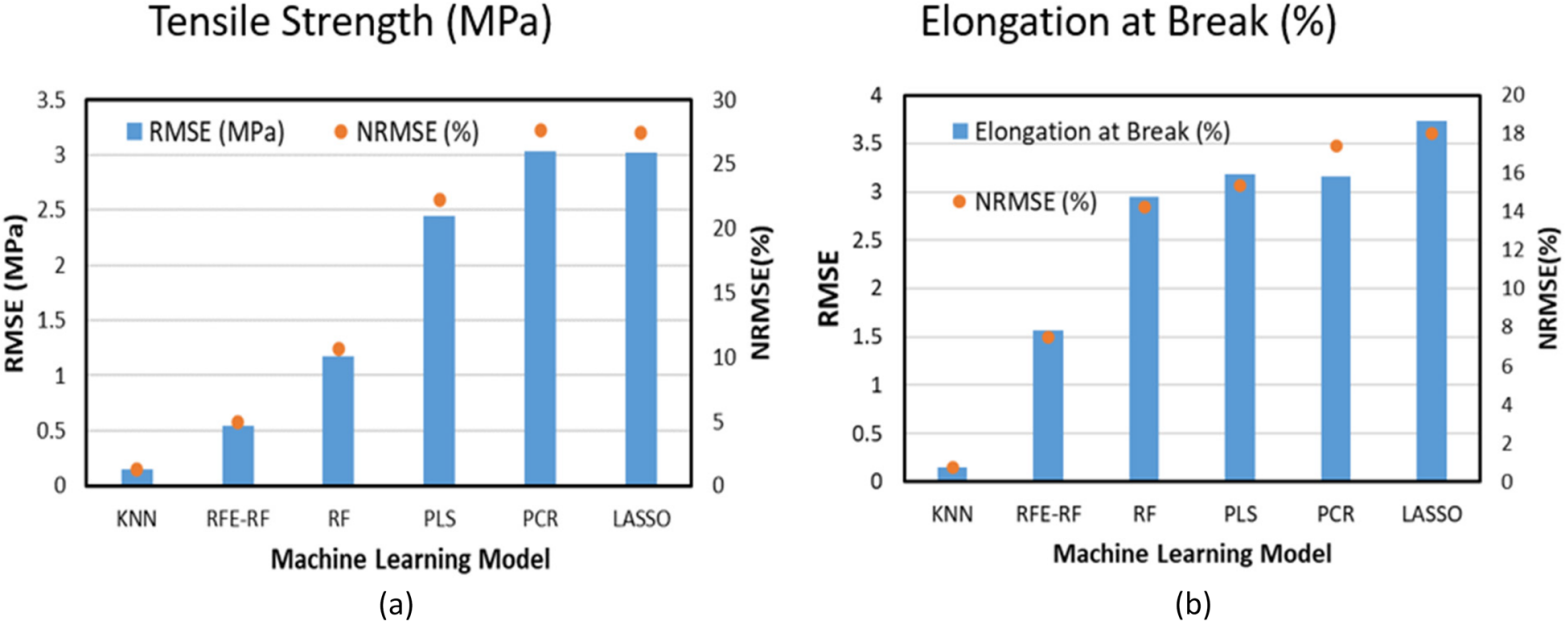
Comparison of ML models' accuracy **a** RMSE to predict tensile strength (MPa), **b** RMSE to predict elongation at break for PLA-INN,ASA samples from in-process data

Figure 8a & b compares the predictive accuracy (RMSE) of the machine learning methods for the prediction of mechanical properties. The non-linear machine learning methods performed well and achieved good predictive accuracy. KNN and RFE-RF (for tensile strength and elongation at break), and RF (for tensile strength only) proved robust enough to achieve good predictive accuracy for different unseen test sets. Linear methods including PLS, PCR and LASSO have previously been reported to achieve good predictive accuracy for drug quantification and drug solid state prediction. However, in this work for the prediction of mechanical properties nonlinear machine learning methods outperformed PLS, PCR and LASSO. Results indicate that in-line NIR coupled with machine learning methods (nonlinear in this case) can predict mechanical properties of drug loaded polymeric matrix over a wide range of processing conditions quite accurately.

 An ablation study was carried out to test the importance of the different sources of input data in achieveing good predictive performance, i.e. separate models were developed using NIR data alone, and also process data alone for the prediction of tensile strength. However, these models did not achieve good predictive accuracy and yielded high RMSE values. Only the combination of  NIR data together with process data achieved good accuracy. Mulrennan et al., 2022 [39] reported similar findings for the prediction of yield stress of the PLA. It should be noted one calibration model was used to assess the predictive ability of the models developed with process data and NIR data separately presented in Table 6 in the Supplementary data, while models presented in Table 14 are validated more robustly using MC-CV.

### Optimal features selected by RFE-RF for the prediction of mechanical properties

**Fig. 9:**
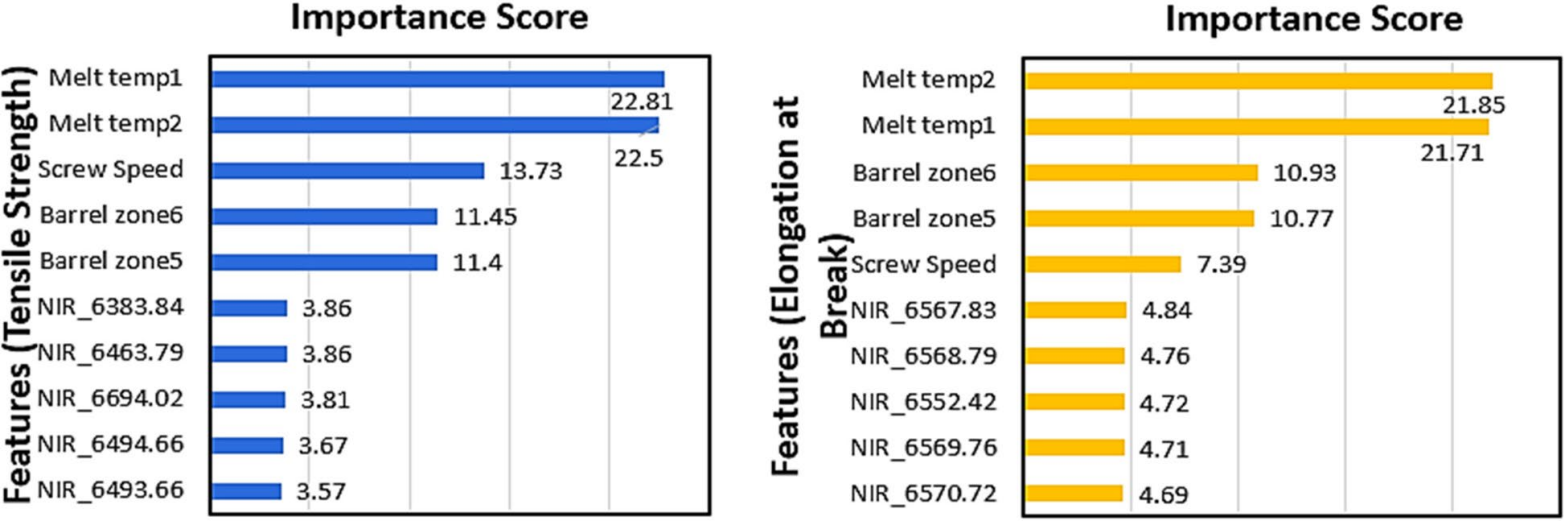
Optimal features and importance score for the prediction of mechanical properties (barrel zone5 represents set temperature in the adapter, barrel zone6 represents set temperature in the die, Melt temp1 and melt temp2 represent melt temperature from die)

Figure 9 shows the features selected by the RFE-RF model along with their importance score for the prediction of tensile strength and elongation at break. For both tensile strength and elongation at break, melt temperatures (captured from the top and bottom of the die at the end of extruder) were highlighted as the top two most important features. From the machine settings, the set temperatures of the adapter and die along with screw speed were also selected as important predictive features for all spectral regions. As NIR spectra are dominated by combination bands and overtone effects, it can be challenging to relate NIR wavenumbers to specific molecular bond activities. For the prediction of tensile strength, most of the wavenumbers were selected from 6400 cm^−1^ region, and couple of wavenumbers from spectral region 6300 cm^−1^, and 6600 cm^−1^. NIR wavenumbers in the selected regions are related to CH_2_ vibrations (first overtone stretching) [81]. For the prediction of elongation at break NIR wavenumbers were only selected from spectral region 6500 cm^−1^. This spectral region is associated with C-H bending and C-H stretching of CH_2_. Stretching and bending vibrations mode of bonds are associated with the movement of atoms withing the polymers’ chain and affect the mechanical properties. For example, C-H stretching of covalent bond affect the tensile strength; stronger the bond (high bond strength) higher would be the tensile strength of the polymer. Similarly the bending mode of vibration is associated with the elasticity of polymers [82]. In previous work for the for the prediction of yield stress of pure PLA from in-process NIR data, NIR wavenumbers in similar regions were also selected [65].

Selected features for the prediction of tensile strength and elongation at break provide important process insights, indicating that the melt temperature in the last two sections of the die has the most significant impact on the mechanical properties of the PLA-ASP product. Therefore, tight melt temperature control for the exit sections of the extruder is important to achieve the desired mechanical properties of PLA-ASP product.

### Selection of the most relevant spectral regions using BiPLS

Backward Interval PLS (BiPLS) was also explored to automate the process of selecting the most relevant NIR spectral regions for prediction of the final product properties for comparison with the manual selection process. For BiPLS, the number of intervals to divide the spectrum into is a tuning parameter, here we varied the number of intervals from 5 to 25 in increments of 5. The best accuracy was achieved with 20 intervals. Table 4 compares the results of the nonlinear machine learning models developed using manually selected spectral region with the models developed using spectral regions selected using BiPLS for the prediction of tensile strength and elongation at break. For the prediction of tensile strength, the models developed with spectral region selected manually achieved better predictive accuracy compared to those developed using BiPLS except for RFE-RF. For tensile strength prediction, RF and KNN yielded high RMSE values for models developed using spectral regions selected by BiPLS. This could be because when models were developed using spectral regions selected by BiPLS, they selected a high number of features and increased model complexity. A slight improvement in accuracy was achieved for BiPLS-RFE-RF as compared to using RFE-RF only. For elongation at break, RF yielded higher RMSE value. KNN achieved good accuracy (RMSE = 0.192) but yielded slightly high RMSE value for models developed using spectral regions selected by BiPLS. In the case of BiPLS-RFE-RF again a slight improvement in the accuracy was achieved.

**Table 4:** Summary of BiPLS-ML to predict tensile strength (MPa)

**Model**	**6100-6700 cm**^-1^	**Spectral region selected by BiPLS (no. of intervals=20)**
**Tensile Strength**	MPa	MPa
RF	1.176	2.255
KNN	0.148	2.609
RFE-RF	0.54	0.42
**Elongation at Break**		
RF	2.944	3.167
KNN	0.153	0.192
RFE-RF	1.566	1.402

To summarise, using a BiPLS step for spectral region selection did not improve the accuracy of machine learning methods significantly. Moreover, using BiPLS step before applying machine learning algorithms requires significant computation and is therefore time-consuming. For this data set, to complete the BiPLS step prior to applying an ML method took an additional twelve minutes. This suggests that selection of spectral regions based on chemical knowledge about wavelengths associated with relevant bond activities is more efficient than automated methods. This also highlights the need to develop a better method to automatically select the spectral regions that contains relevant information.

### Prediction of dissolution profile

Machine learning methods were used to predict the burst release at t = 6 h and the sustained release achieved at t = 96 h. Models were developed using same input features as used to predict the mechanical properties i.e., in-process spectral data, in-process melt temperatures, and process setting data. The performance of each machine learning model was validated using MC-CV with 100 randomly selected unseen test sets. In each iteration (resampling) 70% of the data was randomly selected to train the model and the remaining 30% of the data  was used to test the performance of the model.

As can be seen from Table 5, KNN and RFE-RF models achieved excellent predictive accuracy indicated by low mean RMSE, low mean NRMSE, and high *R* ^2^ values for both time points. The low standard deviation of the RMSE for RFE-RF and KNN is an indication that the models are robust to different training and test sets. RF performed well to predict the initial burst release but gave higher errors for the sustained release predictions. Similar to the case for prediction of mechanical properties, linear machine learning methods including PLS and LASSO performed poorly.

**Table 5:** Comparison of ML models to predict dissolution profile (N__S_= No. of optimal features selected)

**Spectral region 6100-6700 cm**^-1^					
**t=6 hr**	**Mean RMSE**	**SD RMSE**	**Mean NRMSE**	**R**^2^	**SD R**^2^
RF	0.132	0.089	1.55%	0.998	0.004
RFE-RF (N__S_=12)	0.347	0.053	4.02%	0.985	0.0047
KNN	0.558	0.099	6.5%	0.958	0.013
PLS	2.094	0.062	24.7%	0.408	0.044
LASSO	2.139	0.073	25.2%	0.385	0.048
**t=96 hr**	**Mean RMSE**	**SD RMSE**	**Mean NRMSE**	**R**^2^	**SD R**^2^
KNN	0.988	0.155	5.4%	0.96	0.012
RFE-RF (N__S_=10)	1.118	0.173	6.1%	0.97	0.012
RF	3.275	0.154	17.9%	0.811	0.048
PLS	4.758	0.157	26.0%	0.068	0.033
LASSO	5.508	0.182	30.1%	0.106	0.01

For t = 6 h, RFE-RF achieved better predictive accuracy than KNN for all spectral regions. To predict the degree of sustained release at t = 96 h, KNN achieved better predictive accuracy than RFE-RF for all three spectral regions. In the literature KNN has shown good predictive accuracy in similar applications. The performance of a KNN model was compared with other machine learning methods to analyse different aspects of solid dosage forms [83] e.g., for dissolution profile prediction [36], for analysing physical stability of solid dispersion [84], and to analyse defects in solid dosage forms [85]. However, an advantage of using RFE-RF which is a feature selection method over KNN is that RFE-RF provides important process insights by highlighting influential process variables. This information can help to better understand the underlying process factors and relationship of these factors and product quality. Key process variables once identified also can help to control and optimise the HME process to achieve desired final properties.

This work for the first-time reports prediction of drug release profile from in-line data at a range of different processing conditions. Before this, to the best of knowledge, only one study has been reported in which NIR was used ‘at line’. In this work, RFE-RF and K-NN achieved good predictive accuracy (low RMSE), good linearity (close correspondence between predicted and measured values over the range of measurement) and good precision (low standard deviation of errors) for both time points. For example, for t = 6 h, KNN and RFE-RF achieved linearity 95.8%, 98.5%, and standard deviation of errors (as an indication of precision) 9.9% and 5.3%, and for t = 96 h, KNN and RFE-RF achieved linearity 96.3%, 98.1% and precision 14.3%, and 18.4%, respectively. The normalised RMSE (NRMSE) value for both time points is less than 10% for the K-NN and RFE-RF models (see Table 5). The results indicate that it is possible to predict the extent of burst and sustained release of drug from in-line data using machine learning methods with sufficient accuracy.

### Optimum variables selected by RFE-RF for the prediction of dissolution profile

**Fig. 10:**
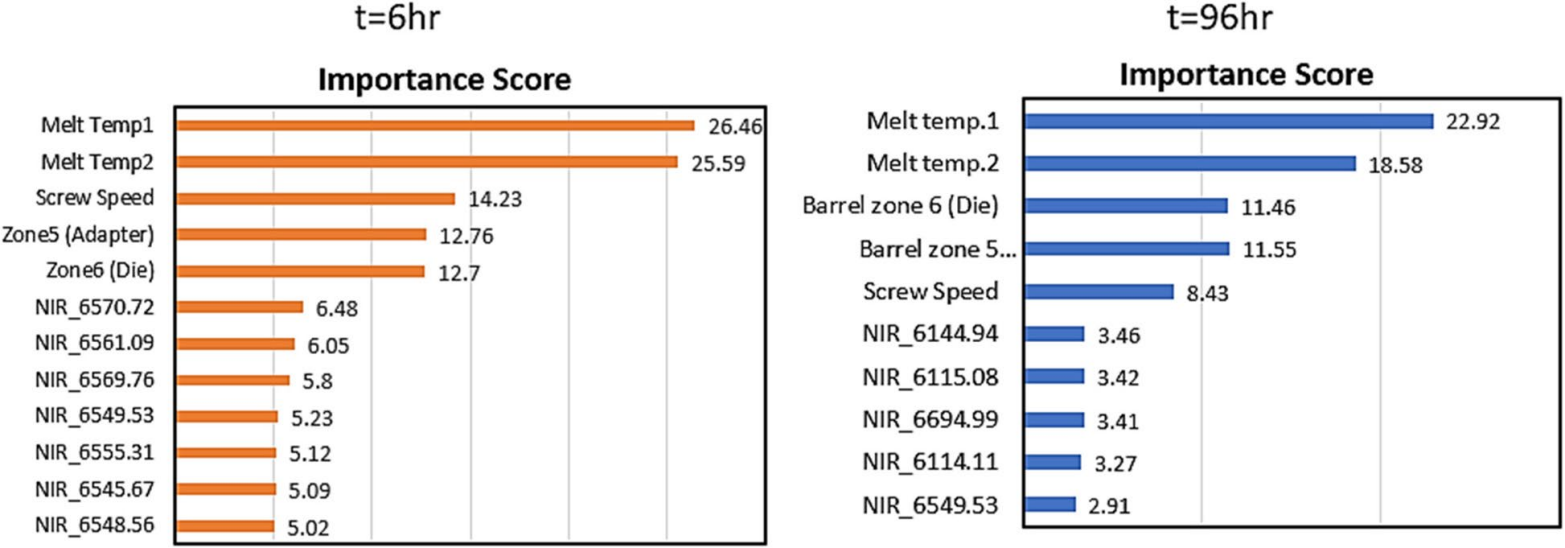
Optimal features and feature importance scores (Dissolution)

Figure 10 shows the optimal features selected and their importance score for prediction of drug release at t = 6 h and t = 96 h. Similar to mechanical properties, the melt temperature and set temperatures from the last sections of the extruder (die and adapter), along with screw speed are selected as the most important variables influencing the dissolution profile. Processing temperature generally has shown to have a significant effect on the release profile as it affects the physical and chemical properties of drug loaded polymeric matrix. If the processing temperature is higher than the melting point of drug (as was the case here for process runs 5–14), it can cause a change in the solid state of the drug from crystalline to amorphous. Solid state of the drug, whether it is more crystalline or amorphous is directly related to dissolution profile of the drug. Drugs in amorphous form have shown better bioavailability and dissolution profile. The solid state of the drug also has significant impact on the solubility and chemical stability of the drug which are two important characteristics studied during development phase [86]. As explained in Sect. 6.1, all the samples processed at different temperatures resulted in different %crystallinity and varied from each other in terms of percentage of drug release as well. So, it is essential to understand the relationship between changes in temperature and its effect on solid state characteristics of polymeric drug delivery systems, and to optimise the temperature to achieve desired release profile [86].

Among NIR wavenumber, wavenumbers mostly were selected from 6100 cm^−1^, 6500 cm^−1^ and some from the 6600 cm^−1^ regions. NIR wavenumbers in these regions are related to CH_2_ stretching vibrations and C-H bending vibrations. For polymer drug delivery systems, NIR wavenumbers are important as these are linked with diffusion of drug molecules, polymer-polymer interactions and polymer chain mobility etc. [87–89]. For example, CH_2_ vibrations provide useful information about polymer chain mobility, which is linked to the diffusion of drug through the polymer matrix. A low stretching frequency indicates high chain mobility and in turn faster drug release. CH_2_ stretching is also linked with the swelling of polymer when it comes in contact with dissolution media [87–89]. Overall, selected features provide important process insight and highlight the importance of stringent temperature control from last two section of extruder (adapter and die) and screw speed as these are the main parameters that effect dissolution properties of PLA-INN,ASA product.

### Conclusion

This work highlighted that processing conditions including screw speed and processing temperature have significant impact on the mechanical and dissolution properties of extruded PLA-INN,ASA. Final properties also depend on concentration of the drug. However, the relationship between processing conditions and final product properties is complex. Thus, methods for predicting the quality during processing are very valuable to help control and optimise the process. The results of this study showed that it is possible to predict final product properties including mechanical properties and dissolution profile of drug loaded PLA matrix from in-line and process settings data analysed using machine learning methods. The performance of all the models was compared by using a robust Monte-Carlo Cross Validation (MC-CV) method, which allows evaluation of the sensitivity of the model to differences in how data is split for training and validation of the model. To predict tensile strength, all nonlinear machine learning methods including RF, KNN, and RFE-RF achieved better predictive accuracy than linear machine learning methods including LASSO, PLS and PCR. However, to predict elongation at break only KNN and RFE-RF achieved good predictive accuracy. Furthermore, the BiPLS method was used to automatically select relevant spectral region for the prediction of mechanical properties of PLA-INN,ASA. Results were compared with the spectral region selected manually based on chemical information. Results showed that selection of spectral regions based on chemical knowledge about wavelengths associated with relevant bond activities wass more efficient than using the BiPLS automated method.

Similarly, all nonlinear methods including RF, KNN and RFE-RF achieved good predictive accuracy to predict the dissolution profile for both time points except for RF at t = 96 h. Overall, KNN and RFE-RF outperformed other methods and proved robust enough to predict different attributes of product for randomly selected unseen test sets. However, an advantage of using RFE-RF over KNN is it provides information about the most influential process variables and this information is useful for process control and optimisation. As highlighted by RFE, melt temperature and process temperature from last two sections of the extruder (adapter and die) along with screw speed were highlighted as most influential variables to predict the mechanical properties and dissolution profile.

This is a first study investigating the potential of in-line NIR for the prediction of drug release. However, as PLA-INN,ASA shows a slow-release profile, in future work a longer-term drug release study will be conducted to provide more time points for prediction. Moreover, more detailed analysis of solid state of the drug will be conducted to relate in-process drug degradation with drug release profile. However, this work highlights the potential of NIR coupled with machine learning methods to predict drug release profile in Hot Melt Extrusion. The results of this work show that NIR coupled with machine learning methods can potentially be used for real-time prediction of mechanical properties and dissolution profile. This can help to rapidly identify process issues and take immediate corrective action if the product is going out of specifications, saving materials, cost, and time.

## Supplementary Information

Supplementary Material 1.

## Data Availability

Model codes are provided in the supplementary material, further data is available from the corresponding author on request.
