# Supplementary material for: In-line NIR coupled with machine learning to predict mechanical properties and dissolution profile of PLA-Aspirin
Source: Funct Compos Mater. 2024 Oct 8;5(1):14. doi: 10.1186/s42252-024-00063-5 (PMC11461551; doi:10.1186/s42252-024-00063-5)
Supplement: Supplementary file 1 — Supplementary Material 1. [file 42252_2024_63_MOESM1_ESM.docx]

# **DSC Curves**

**
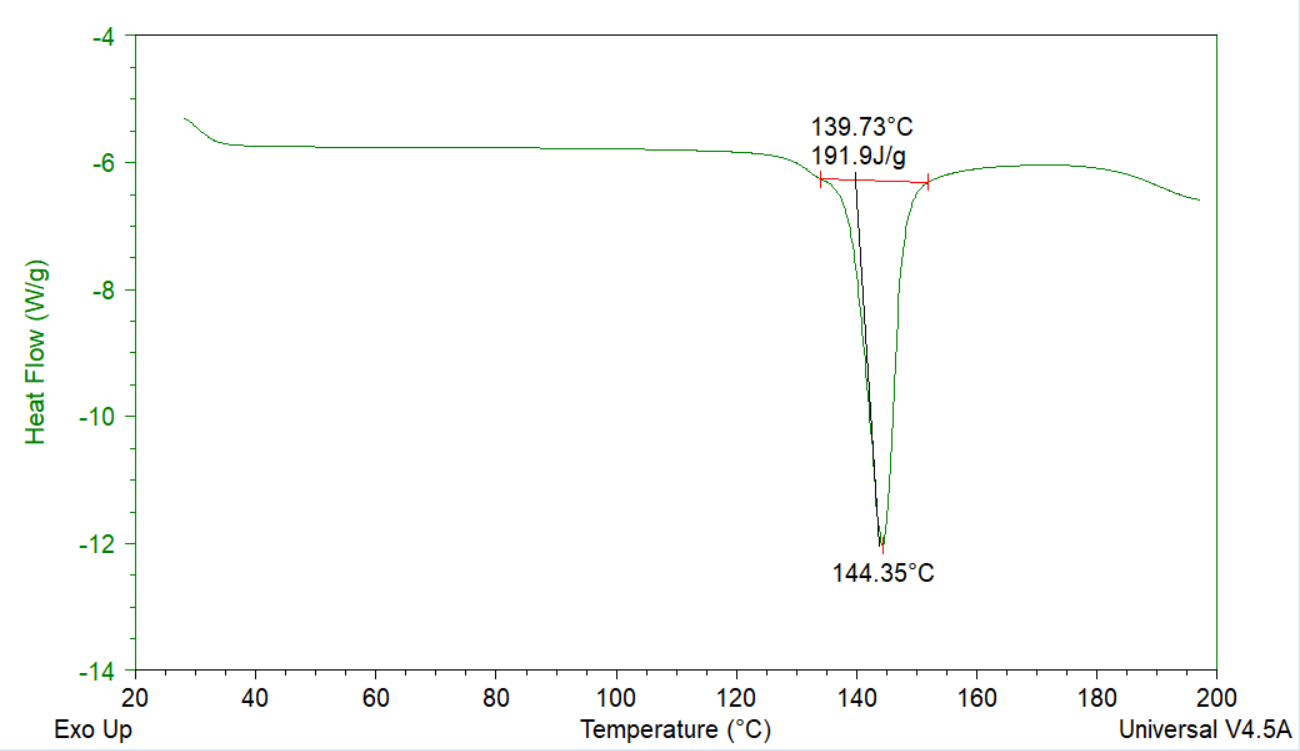
**

Pure Aspirin Sample


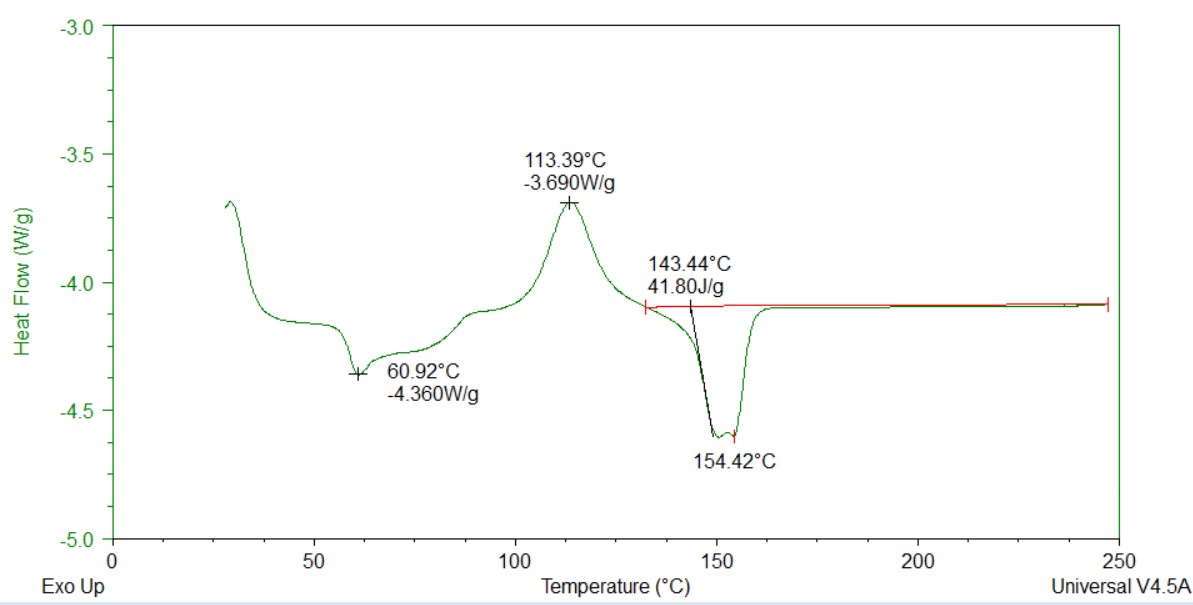


Pure PLA (6 Hz)


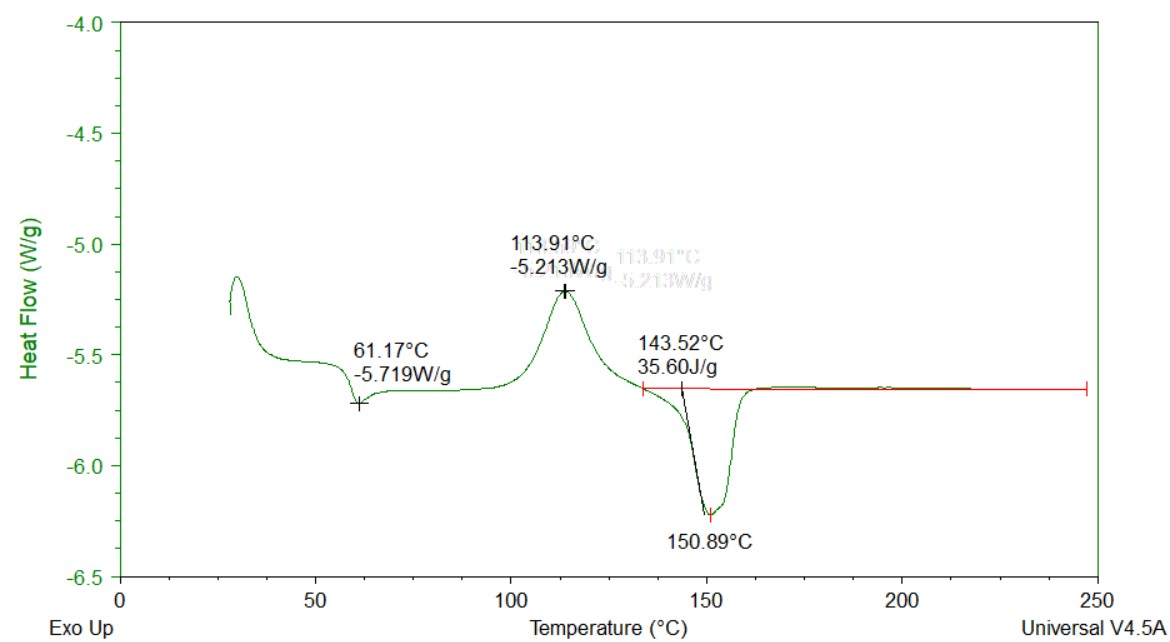


Pure PLA (8 Hz)


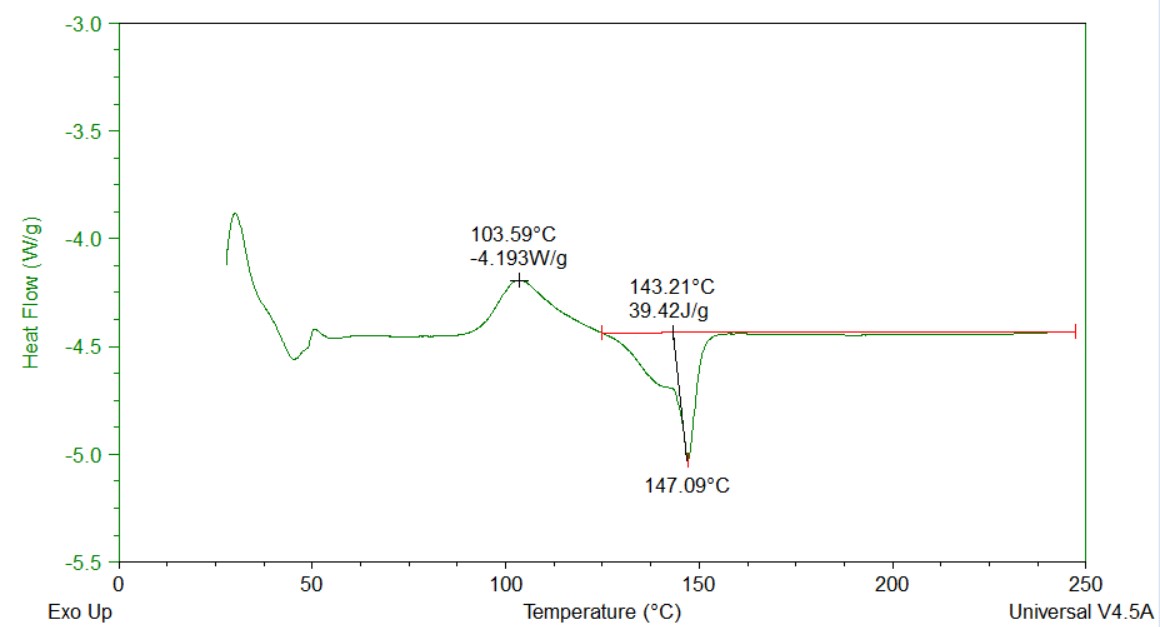


PLA-ASP sample-1


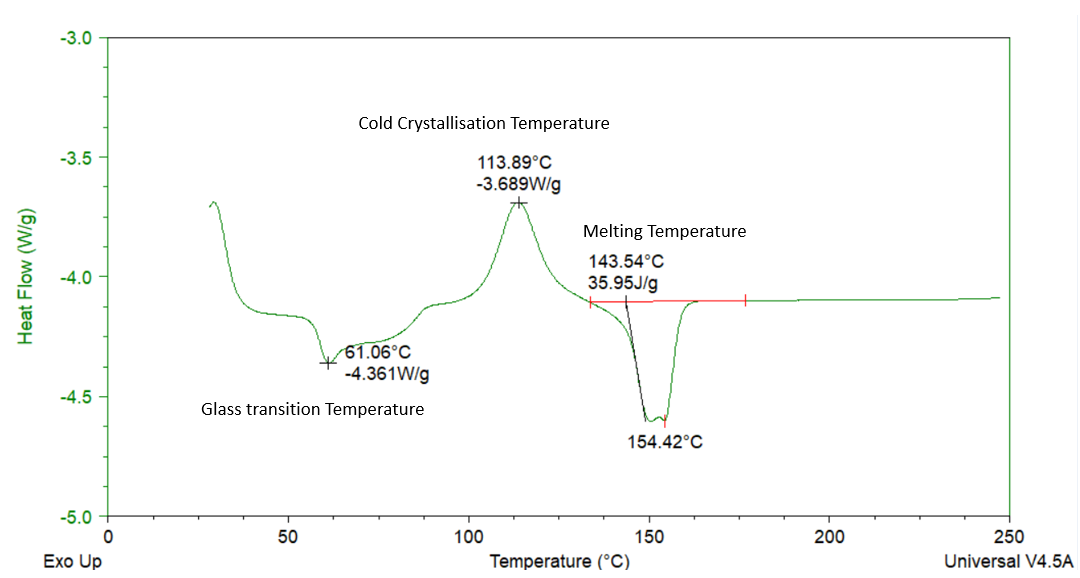


PLA-ASP sample-2

**
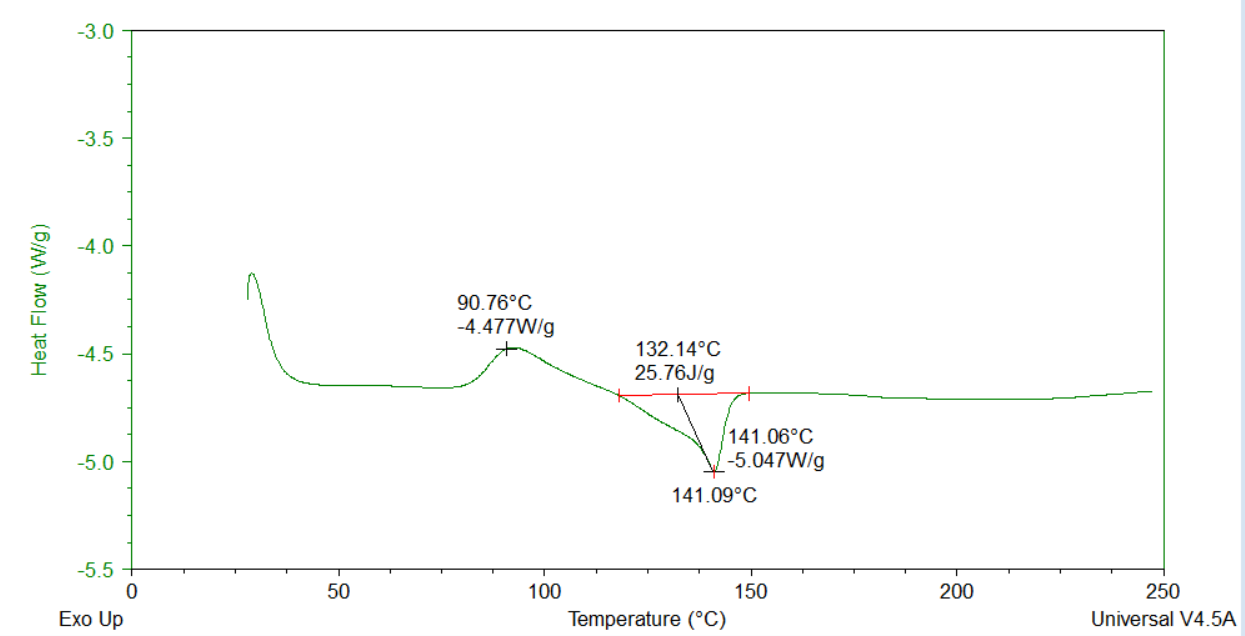
**

PLA-ASP sample-3

**
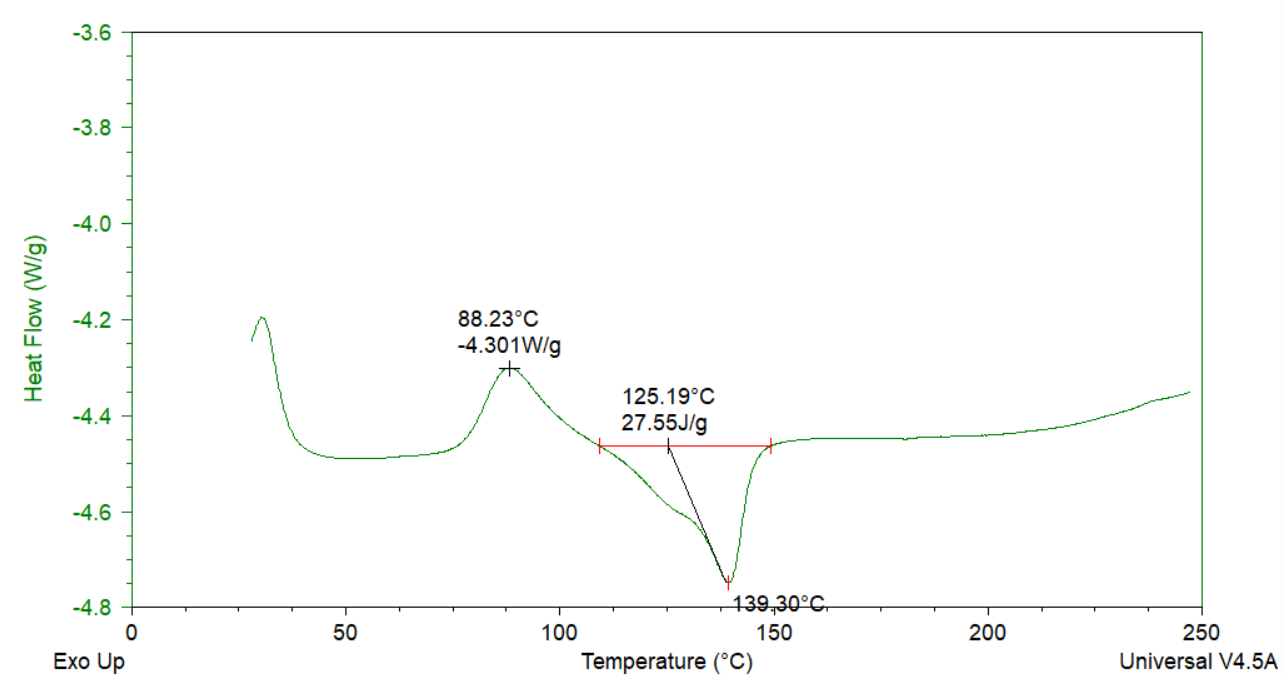
**

PLA-ASP sample-4

**
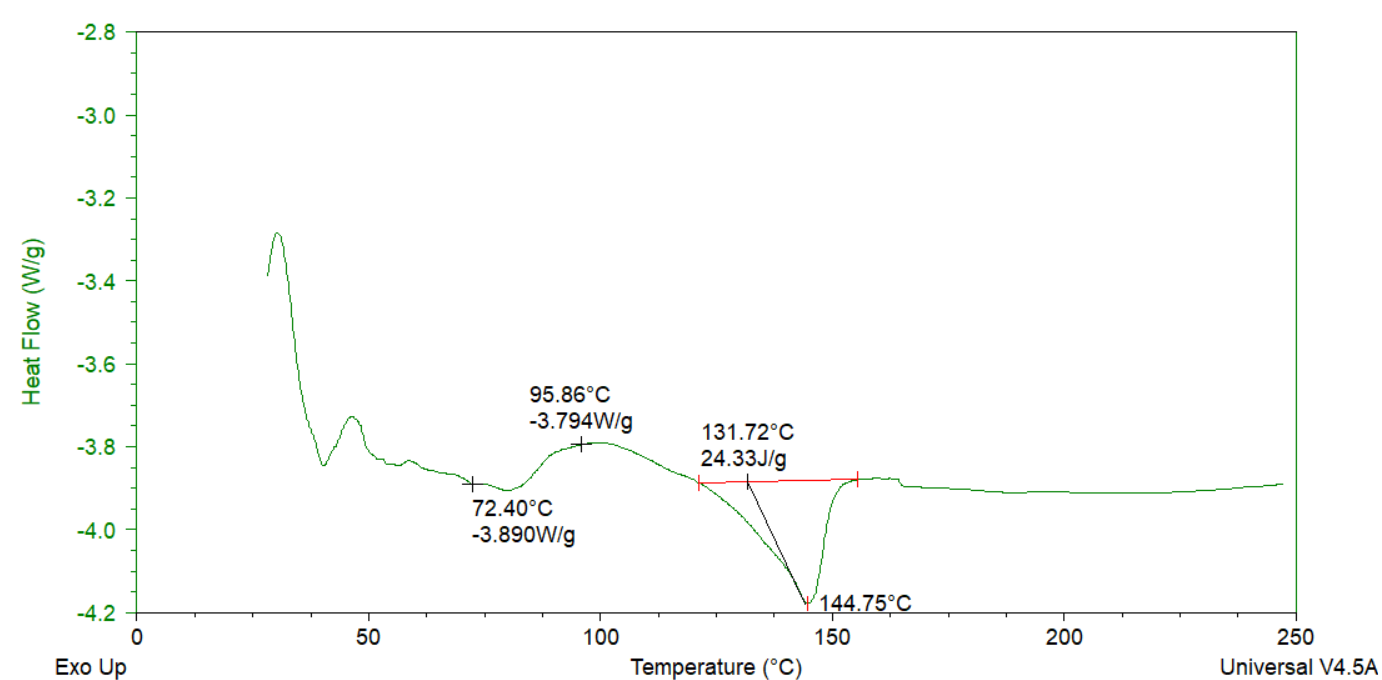
**

PLA-ASP sample-5

**
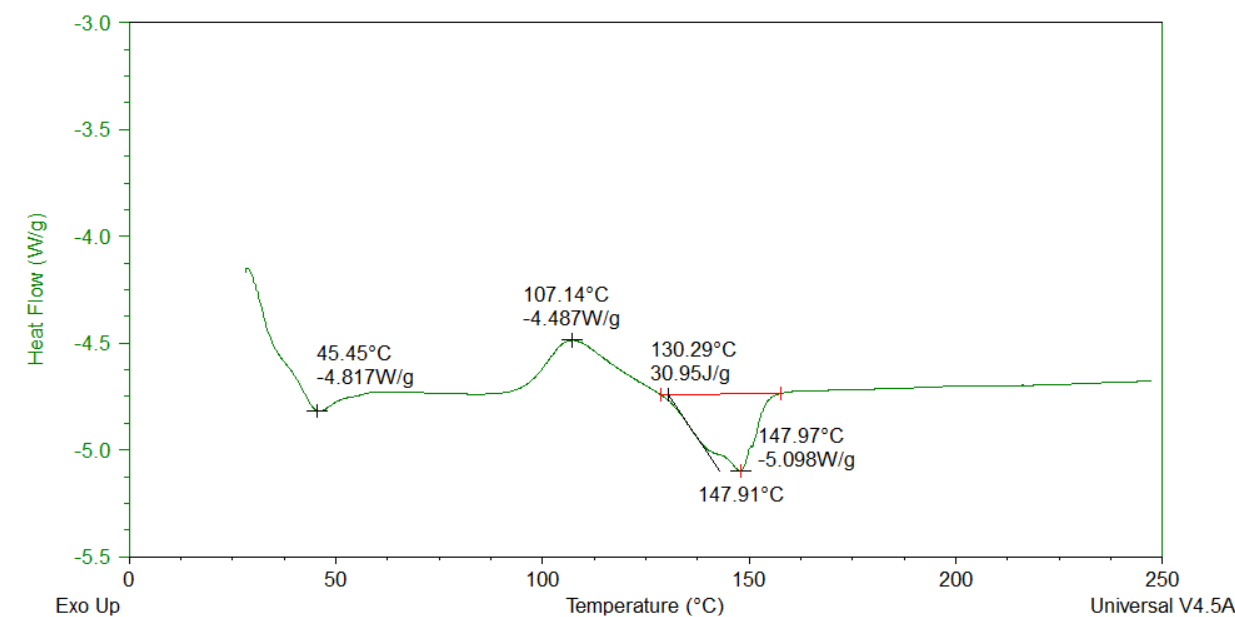
**

PLA-ASP sample-6

**
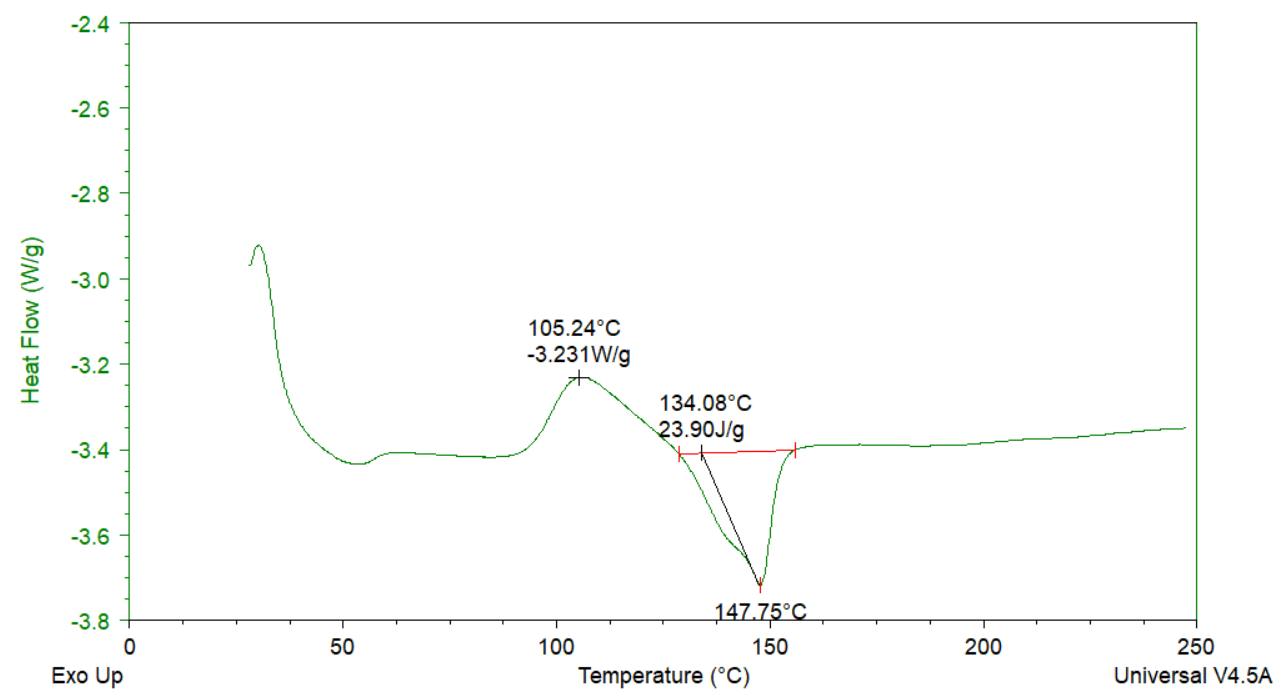
**

PLA-ASP sample-7

**
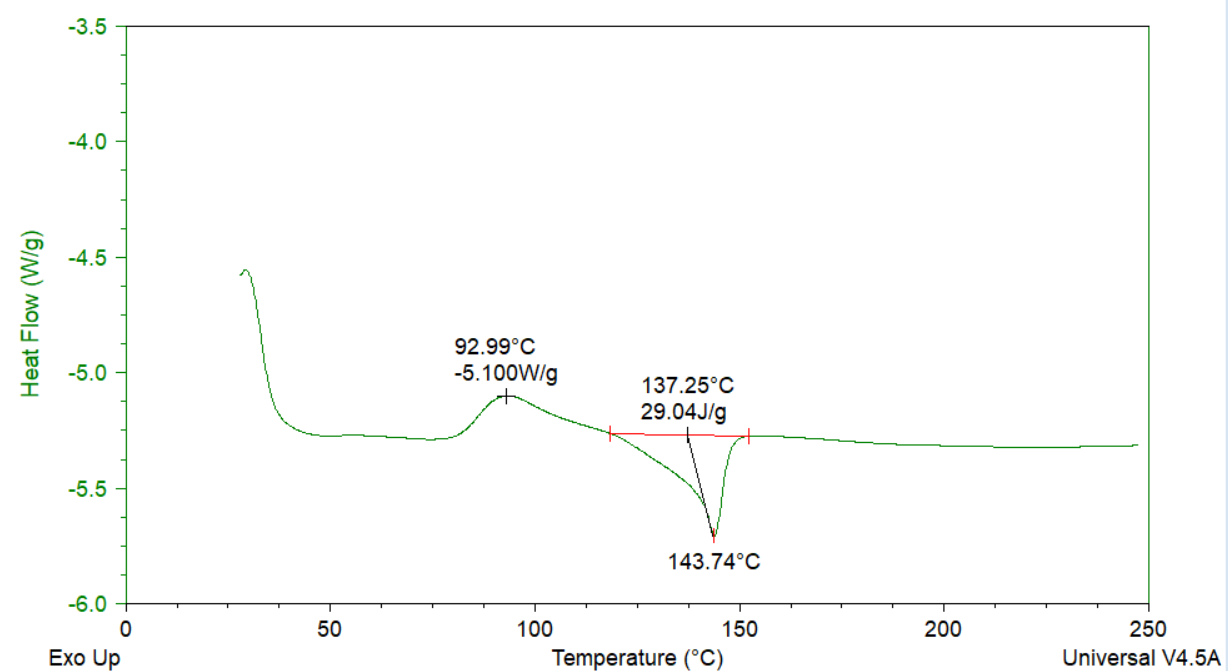
**

PLA-ASP sample-8

**
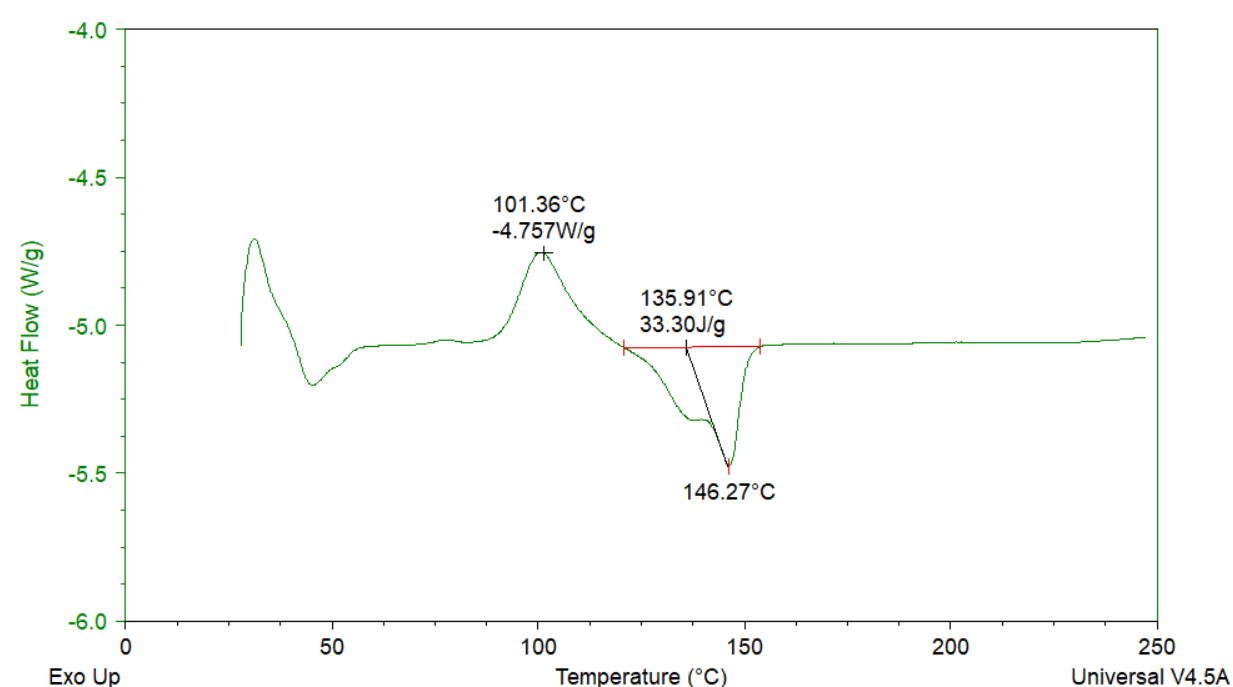
**

PLA-ASP sample-9

**
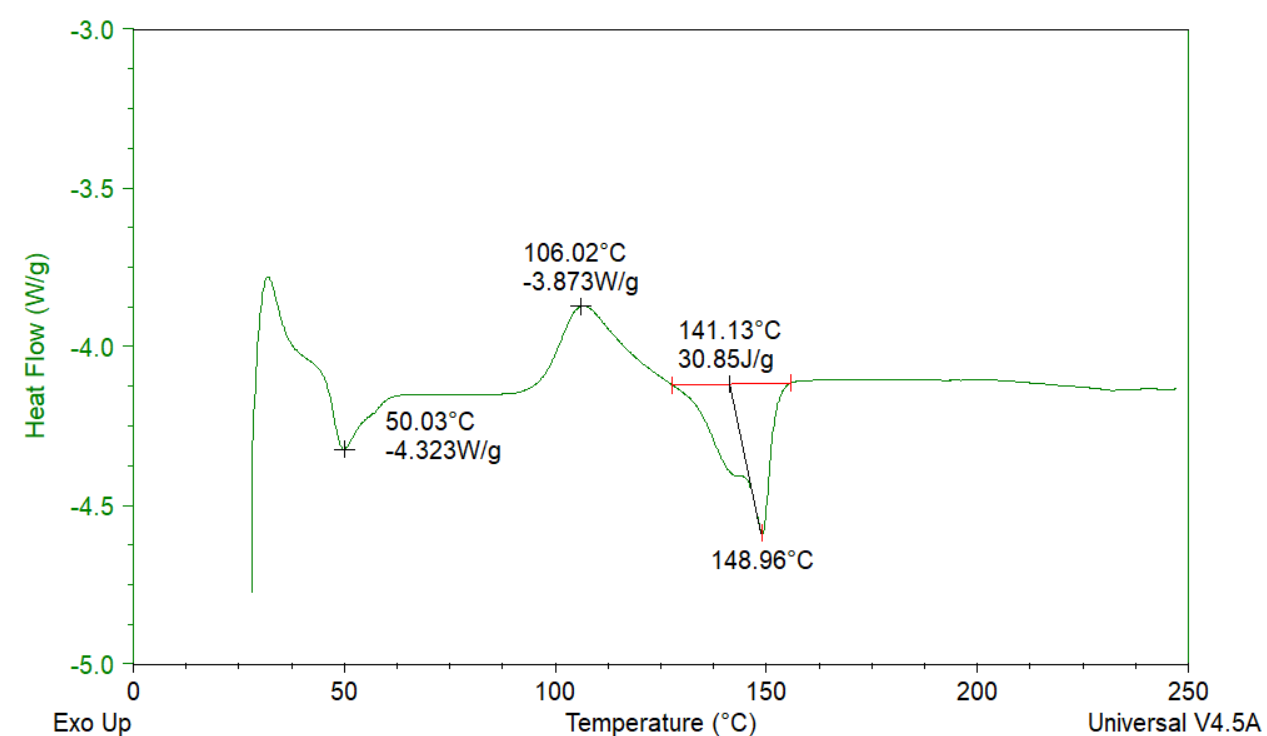
**

PLA-ASP sample-10

**
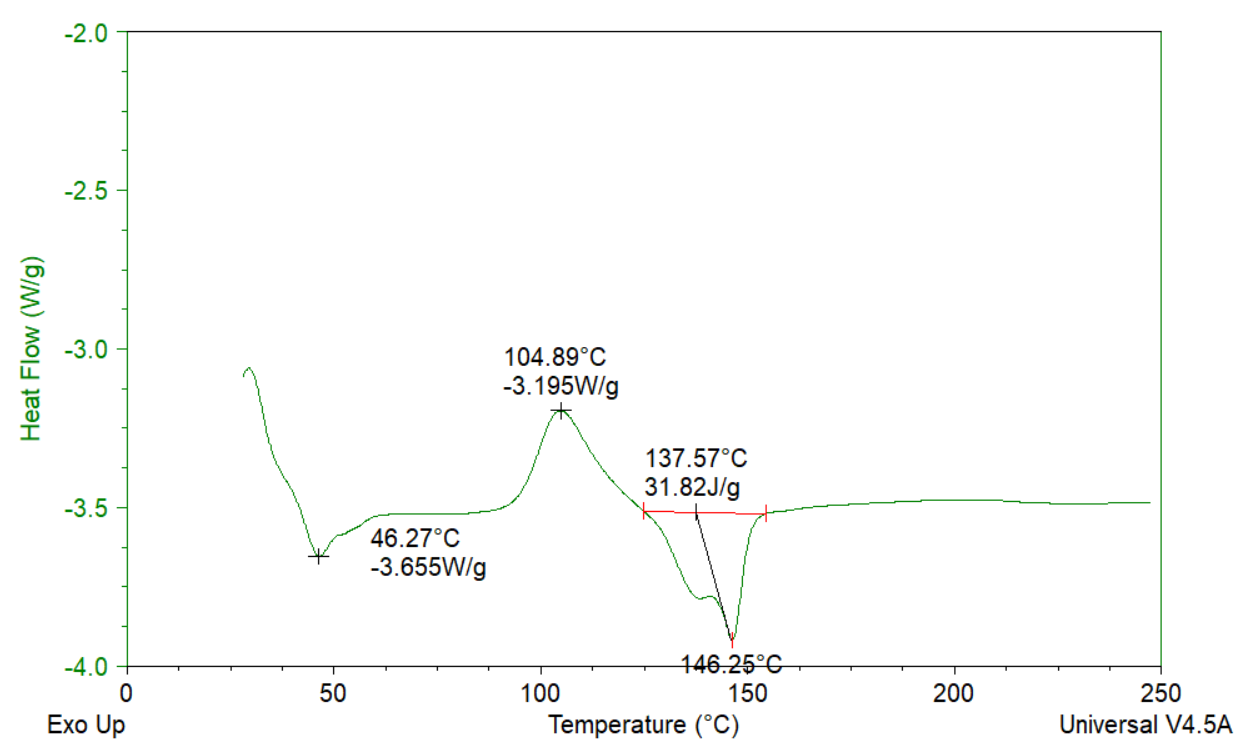
**

PLA-ASP sample-11

**
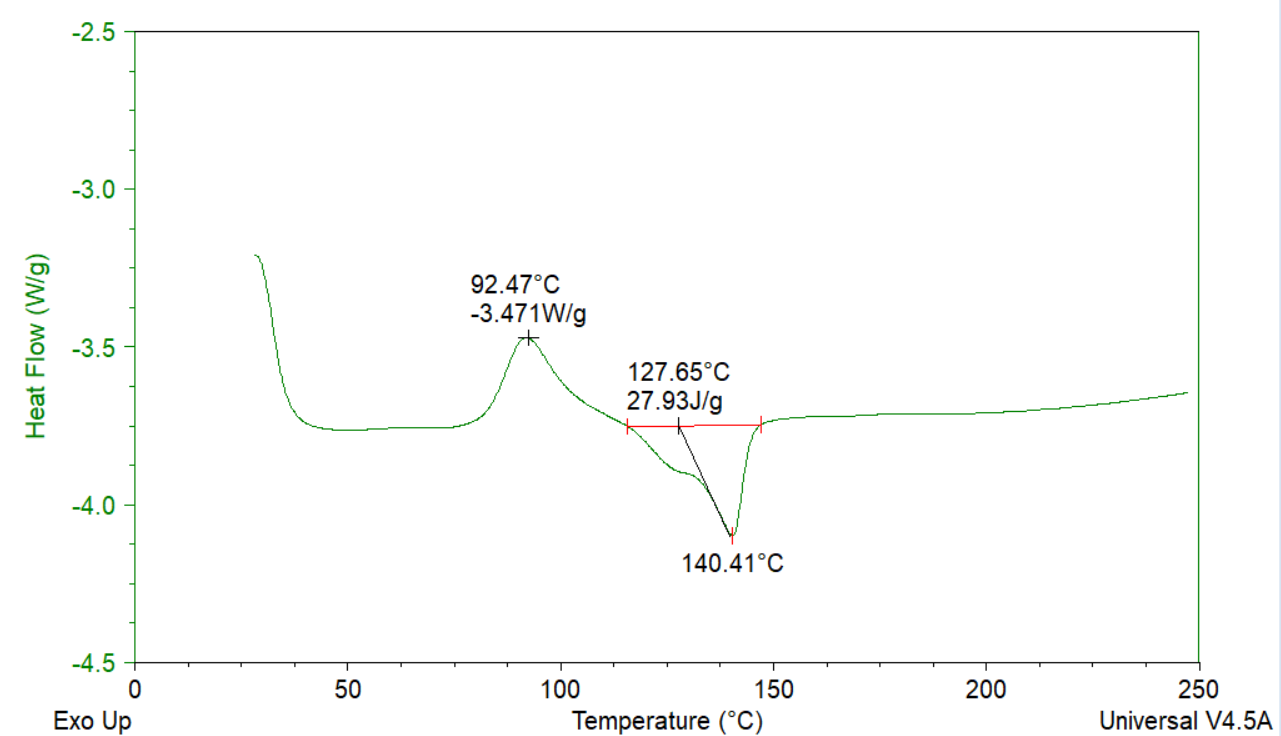
**

PLA-ASP sample-12

**
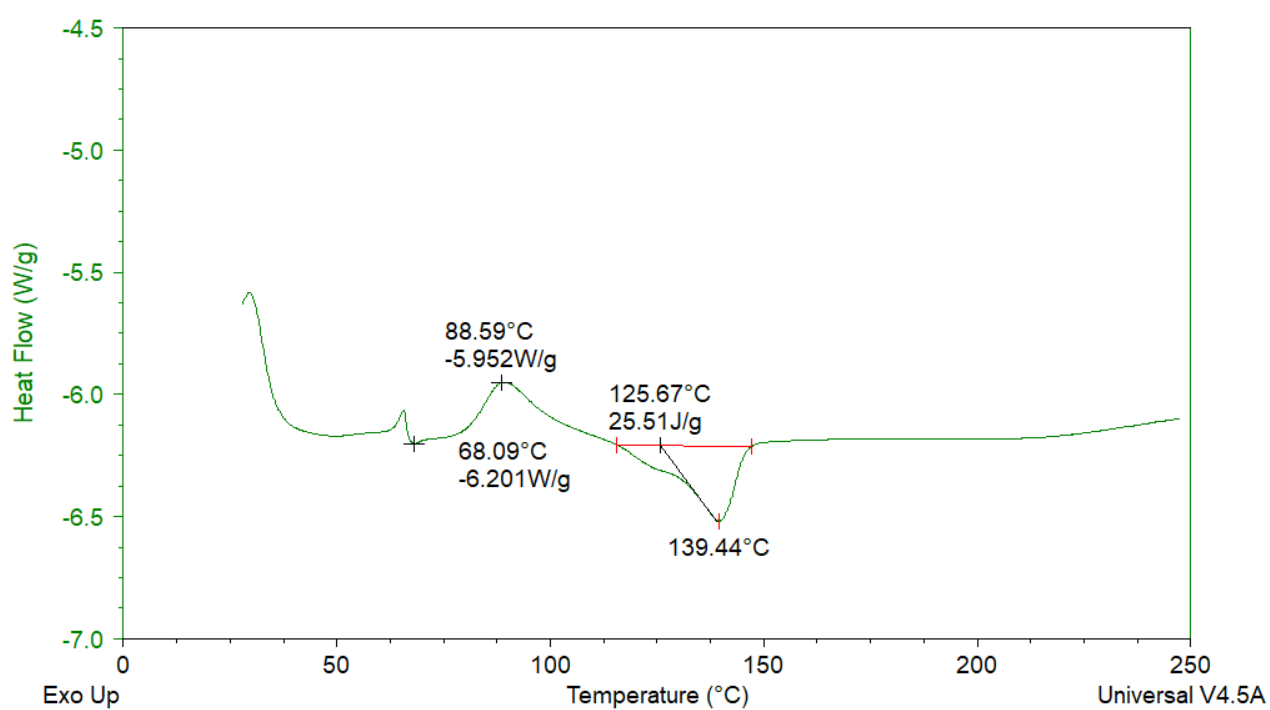
**

PLA-ASP sample-13

**
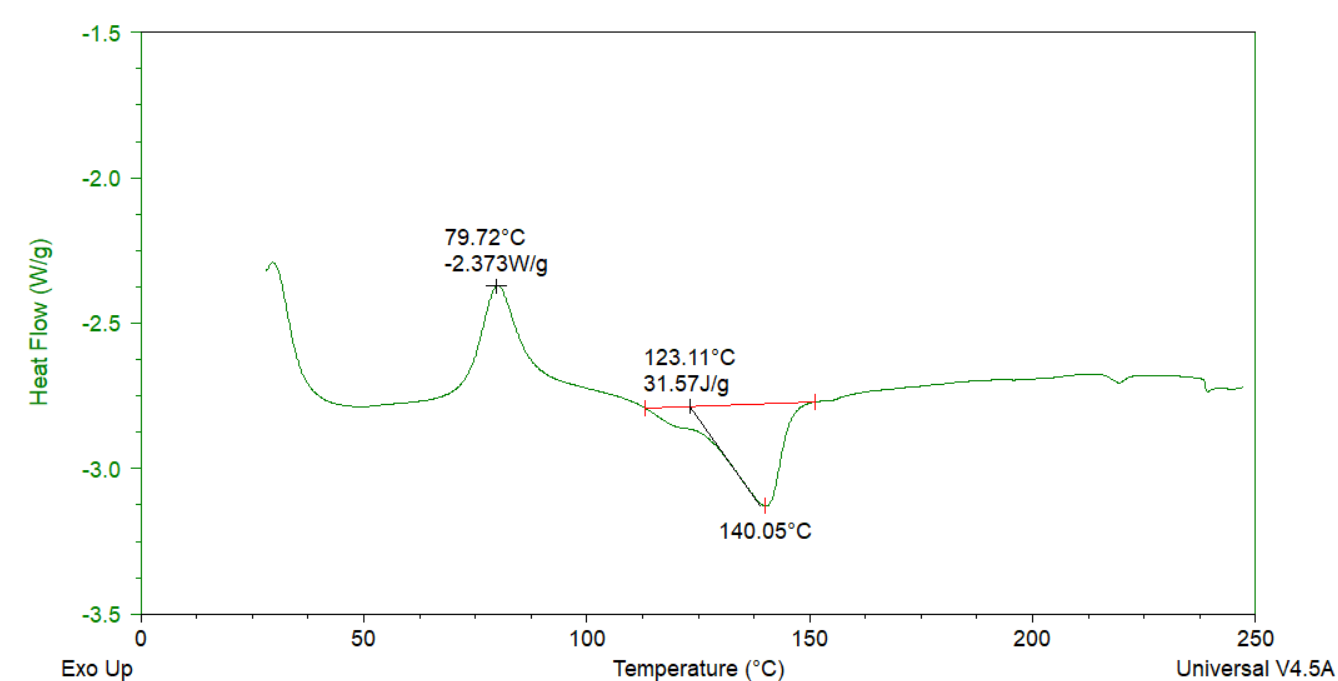
**

PLA-ASP sample-14

Figure 10. DSC curves of PLA-Aspirin samples for process run 1-14.

Table 6 Comparison of model results

| Tensile Strength (MPa) | |
| --- | --- |
| **Process settings data only (One calibration model)** | |
| **RMSE (MPa)** | |
| PLS | 2.727 |
| RF | 2.557 |
| KNN | 3.238 |
| Bagging | 3.279 |
| LASSO | 3.192 |
| **NIR data only (One calibration model)** | |
| PLS | 2.1 |
| RF | 1.161 |
| KNN | 2.686 |
| Bagging | 1.788 |
| LASSO | 2.446 |
| **Combining process data with NIR data (70:30 Train-Test split)** | |
| PLS | 2.04 |
| RF | 0.466 |
| Bagging | 0.273 |
| KNN | 0.124 |
| LASSO | 3.051 |

# **R script**

#NIR Data

library(grid)

library(xml2)

library("hyperSpec")

library("lubridate")

library(randomForest)

library("ggplot2")

library("lattice")

library("AppliedPredictiveModeling")

library("caret")

library("glmnet")

library("mlbench")

library("pls")

library("tidyverse")

library(Metrics)

library(xlsx)

#######5000-6700#####

setwd("C:/Users/S00202658/OneDrive - Institute of Technology Sligo/Desktop/data/NIR_18-05-2022")

set.seed(1010)

filelist <- list.files(pattern = "*.txt")

buffer <- matrix (scan(filelist [1]), ncol = 2, byrow = TRUE)

wavelength <- buffer [,1]

spcdata <- matrix (ncol = nrow(buffer), nrow = length(filelist))

spcdata [1,] <- buffer[,2]

for (f in seq(along = filelist)[-1]){

buffer <- matrix (scan(filelist [f], quiet = TRUE), ncol =2, byrow = TRUE)

spcdata[f,] <- buffer [,2]

}

rm(f,buffer)

cat (length(filelist), "files imported.")

cat (" Wavelength range =", wavelength[1], "-",

(wavelength[1]-(length(wavelength)-1)))

nirts <- gsub("_", ":", filelist)

nirts <- gsub(".txt", "", nirts)

nirts <- period_to_seconds(hms(nirts))

init_time <- period_to_seconds(hms("11:00:00"))

qt <- c(period_to_seconds(hms("11:20:43")) - init_time,

period_to_seconds(hms("11:38:00")) - init_time,

period_to_seconds(hms("12:03:13")) - init_time,

period_to_seconds(hms("12:21:07")) - init_time,

period_to_seconds(hms("12:45:34")) - init_time,

period_to_seconds(hms("12:58:35")) - init_time,

period_to_seconds(hms("13:20:18")) - init_time,

period_to_seconds(hms("13:28:50")) - init_time,

period_to_seconds(hms("14:14:43")) - init_time,

period_to_seconds(hms("14:33:46")) - init_time,

period_to_seconds(hms("14:58:09")) - init_time,

period_to_seconds(hms("15:21:10")) - init_time,

period_to_seconds(hms("15:57:29")) - init_time,

period_to_seconds(hms("16:17:44")) - init_time)

qt <- as.data.frame(qt)

nirts <- nirts - init_time

nirts <- data.frame(nirts)

spcdata <- data.frame(spcdata)

spcdata$time <- nirts[,1]

spcdata$ID <- c((sprintf("one",seq(2:31))),

(sprintf("two", seq(32:85))),

(sprintf("three", seq(86:107))),

(sprintf("four", seq(108:129))),

(sprintf("five", seq(130:168))),

(sprintf("six", seq(169:218))),

(sprintf("seven", seq(219:246))),

(sprintf("eight", seq(247:310))),

(sprintf("nine", seq(311:365))),

(sprintf("ten", seq(366:427))),

(sprintf("eleven", seq(428:497))),

(sprintf("twelve", seq(498:566))),

(sprintf("thirteen", seq(567:617))),

(sprintf("fourteen", seq(618:629))))

spcdata_process_runs <- spcdata

spcdata <- new("hyperSpec", spc = spcdata_process_runs[, 1:3633],

wavelength = wavelength)

plot(spcdata, spc.nmax=290, col=c(1,2,3,4,5,6,7))

spcdataA <- spcdata

spcdataA [[]] <- apply (spcdata [[]] , 1:2 , function (x) 2-log (x))

plot(spcdataA, col=c(1,2,3,4,5,6,7))

spctrim <- spcdataA[,,5000:6700]

plot(spctrim, col=c(1,2,3,4,5,6,7))

spctrim.msc <- spctrim

spctrim.msc[[]] <- msc(spctrim[[]])

plot(spctrim.msc, col=c(1,2,3,4,5,6,7))

bl <- spc.fit.poly.below(spctrim.msc)

spctrim.msc.bl <- spctrim.msc - bl

plot(spctrim.msc.bl, col=c(1,2,3,4,5,6,7))

spcdata_may22 <- as.data.frame(spctrim.msc.bl$spc)

spcdata_may22$ID <- spcdata_process_runs$ID

spcdata_may22$time <- spcdata_process_runs$time

spcdata_may22$date <- dmy("18-05-2022")

spcdata_may22$time <- spcdata_may22$time + init_time

spcdata_may22$timestamp <- seconds_to_period(spcdata_may22$time)

spcdata_may22$timestamp <- spcdata_may22$timestamp + dmy("18-05-2022")

mydata_one<-spcdata_may22

**Modelling of data**

set.seed(1010)

library(readxl)

X6100_6700 <- read_excel("C:/Users/munir/OneDrive - Atlantic TU/Desktop/data/Images and data sets/6100-6700.xlsx")

mydata_three<-X6100_6700

set.seed(1010)

library(readxl)

Process_conditions <- read_excel("C:/Users/munir/OneDrive - Atlantic TU/Desktop/data/Process conditions.xlsx")

mydata_three<- Process_conditions

#####Tensile Strength####

set.seed(1010)

library(readxl)

Mech_properties <- read_excel("C:/Users/munir/OneDrive - Atlantic TU/Desktop/data/Mech_properties.xlsx")

Mech_properties<-data.frame(Mech_properties)

mydata_three <- merge(mydata_three, Mech_properties, by="ID")

movetolast <- function(data, move) {

data[c(setdiff(names(data), move), move)]

}

mydata_three <- movetolast(mydata_three, c("Tensile.Strength", "ID", "time", "date", "timestamp"))

**Prediction of Breaking Elongation**

set.seed(1010)

library(readxl)

X6100_6700 <- read_excel("C:/Users/Munir/OneDrive - Atlantic TU/Desktop/data/Images and data sets/6100-6700.xlsx")

mydata_three<-X6100_6700

set.seed(1010)

library(readxl)

Process_conditions <- read_excel("C:/Users/Munir/OneDrive - Atlantic TU/Desktop/data/Process conditions.xlsx")

mydata_three<-merge(mydata_three, Process_conditions, by="ID")

######MC-CV 6100-6700 BE#####

set.seed(1010)

library(readxl)

Breaking_elongation <- read_excel("C:/Users/Munir/OneDrive - Atlantic TU/Desktop/data/Breaking elongation.xlsx")

Breaking_elongation<-data.frame(Breaking_elongation)

mydata_three <- merge(mydata_three, Breaking_elongation, by="ID")

movetolast <- function(data, move) {

data[c(setdiff(names(data), move), move)]

}

mydata_three <- movetolast(mydata_three, c("X.Breaking.elongation", "ID", "time", "date", "timestamp"))

###############LASSO BE 6100-6700####

set.seed(1010)

mydata_three<-mydata_three[,1:611]

set.seed(1010)

custom<-trainControl(method = "LGOCV", number = 100,p=0.7 ,verbose = TRUE)

lasso_model_BE_three<- train(mydata_three[,1:610], mydata_three[,611],

method = "glmnet",

trControl = custom,

tuneGrid = expand.grid(alpha = 1, lambda = seq(0.001, 0.1, by = 0.001)))

lasso_model_TS_mean<-lasso_model_BE_three$resample$RMSE

lasso_model_TS_rsq<-lasso_model_BE_three$resample$Rsquared

mean(lasso_model_TS_mean)

sd(lasso_model_TS_mean)

mean(lasso_model_TS_rsq)

sd(lasso_model_TS_rsq)

predictors(lasso_model_BE_three)

3.731/(max(mydata_three$X.Breaking.elongation)-min(mydata_three$X.Breaking.elongation))

##MC-CV, selected=139, mean rmse=3.731, sd=0.223, mean nrmse=0.180,mean rsq=0.212, sd rsq=0.035....

######MC-CV rfe 6100-6700 BE####

set.seed(1010)

custom<-rfeControl(functions = rfFuncs, method = "LGOCV",p=0.7 ,number = 100, verbose = TRUE)

train_model<- rfe(mydata_three[,1:610], mydata_three[,611],

sizes = c(10:100), rfeControl = custom)

train_model$bestSubset

af<-train_model$resample$RMSE

af<-data.frame(af)

mean(af)

sd(af)

am<-train_model$resample$Rsquared

mean(am)

sd(am)

train_model$optVariables

#[1] "Melt temp2 21.85" "Melt temperature1 21.71" "zone6 10.93" "zone5 10.77" "scew_speed 7.39"

#[6] "6568.79 (4.76)" "6552.42 (4.72)" "6567.83 (4.84)" "6569.76 (4.71)" "6570.72(4.69)"

###mean rmse=1.566, sd of rmse=0.45, mean rsq=0.875, sd of r2=0.065

varImp(train_model)

write.xlsx(af, file = "%BE MC_CV 6100-6700 RMSE.xlsx")

#####KNN 6100-6700 BE#####

set.seed(1010)

knn_three_train_be<-train(X.Breaking.elongation~.,data=mydata_three[,1:611],method="knn",

trControl=trainControl(method = "LGOCV", number = 100,verboseIter = TRUE),

tuneGrid=expand.grid(k=seq(2,15,by=1)))

ssv<-knn_three_train_be$resample$RMSE

vvs<-knn_three_train_be$resample$Rsquared

mean(ssv)

sd(ssv)

mean(vvs)

sd(vvs)

#MC-CV, k=5, mean rmse=0.153, rmse sd=0.0326, rsq mean=0.998,sd rsq=0.0005

set.seed(1010)

rf_three_train<-train(X.Breaking.elongation~.,data=mydata_three[,1:611],

method="rf", trControl=trainControl(method = "LGOCV", number = 100,p=0.7,savePredictions = "all", verboseIter = TRUE),

tuneGrid=expand.grid(mtry=seq(350,500,by=50)))

r5<-rf_three_train$resample$RMSE

r6<-rf_three_train$resample$Rsquared

mean(r5)

sd(r5)

mean(r6)

sd(r6)

####mean rmse=2.944, sd of rmse=0.439, mean rsq=0.488, sd of rsq=0.081

##############6100-6700 BE MC-CV####

set.seed(1010)

pls_BE_train_three<-train(X.Breaking.elongation~.,data=mydata_three,method="pls",

trControl=trainControl(method = "LGOCV", number = 100,p=0.7,verboseIter = TRUE))

R101<-pls_BE_train_three$resample$RMSE

R202<-pls_BE_train_three$resample$Rsquared

mean(R101)

sd(R101)

mean(R202)

sd(R202)

#mean rmse=3.177, sd rmse=0.271, mean rsq=0.396, sd r2=0.030, nrmse=0.153

3.177/(max(mydata_three$X.Breaking.elongation)-min(mydata_three$X.Breaking.elongation))

#################PCR BE 6100-6700##########

set.seed(1010)

pcr_three_train_be<-train(X.Breaking.elongation~.,data=mydata_three,

method="pcr", trControl=trainControl(method = "LGOCV", number = 100,p=0.7,

savePredictions = "all", verboseIter = TRUE))

rmean_three_be<-pcr_three_train_be$resample$RMSE

rsq_three_be<-pcr_three_train_be$resample$Rsquared

mean(rmean_three_be)

sd(rmean_three_be)

mean(rsq_three_be)

sd(rsq_three_be)

#ncomp=3, mean rmse=3.614,sd rmse=0.354, mean nrmse=0.174, mean rsq=0.213,sd rsq=0.033

3.614/(max(mydata_three$X.Breaking.elongation)-min(mydata_three$X.Breaking.elongation))

**Prediction of Tensile Strength**

set.seed(1010)

library(readxl)

Process_conditions <- read_excel("C:/Users/Munir/OneDrive - Atlantic TU/Desktop/data/Process conditions.xlsx")

mydata_three<-merge(mydata_three, Process_conditions, by="ID")

#####Tensile Strength####

set.seed(1010)

library(readxl)

Mech_properties <- read_excel("C:/Users/munir/OneDrive - Atlantic TU/Desktop/data/Mech_properties.xlsx")

Mech_properties<-data.frame(Mech_properties)

mydata_three <- merge(mydata_three, Mech_properties, by="ID")

movetolast <- function(data, move) {

data[c(setdiff(names(data), move), move)]

}

mydata_three <- movetolast(mydata_three, c("Tensile.Strength", "ID", "time", "date", "timestamp"))

###############LASSO TS 6100-6700####

set.seed(1010)

mydata_three<-mydata_three[,1:611]

set.seed(1010)

custom<-trainControl(method = "LGOCV", number = 100,p=0.7 ,verbose = TRUE)

lasso_model_TS_three<- train(mydata_three[,1:610], mydata_three[,611],

method = "glmnet",

trControl = custom,

tuneGrid = expand.grid(alpha = 1, lambda = seq(0.001, 0.1, by = 0.001)))

lasso_model_TS_mean<-lasso_model_TS_three$resample$RMSE

lasso_model_TS_rsq<-lasso_model_TS_three$resample$Rsquared

mean(lasso_model_TS_mean)

sd(lasso_model_TS_mean)

mean(lasso_model_TS_rsq)

sd(lasso_model_TS_rsq)

predictors(lasso_model_TS_three)

3.019/(max(mydata_three$Tensile.Strength)-min(mydata_three$Tensile.Strength))

##MC-CV, selected=136, mean rmse=3.019, sd=0.0965, mean nrmse=0.274,mean rsq=0.094, sd rsq=0.032....

######MCCV rfe 6100-6700 TS####

set.seed(1010)

custom<-rfeControl(functions = rfFuncs, method = "LGOCV",p=0.7 ,number = 100, verbose = TRUE)

train_model<- rfe(mydata_three[,1:610], mydata_three[,611],

sizes = c(10:100), rfeControl = custom)

train_model$bestSubset

af<-train_model$resample$RMSE

mean(af)

sd(af)

am<-train_model$resample$Rsquared

mean(am)

sd(am)

#selected=10, mean=0.540, sd=0.086, mean rsq=0.979, sd rsq=0.006...TS

#Melt temp1 22.81

#Melt temp2 22.5

#Screw Speed 13.73

#Barrel zone5 11.4

#Barrel zone6 11.45

#NIR_6383.84 3.86

#NIR_6694.02 3.81

#NIR_6463.79 3.86

#NIR_6493.66 3.57

#NIR_6494.66 3.67

######MC-CV 6100-6700 RF TS########

set.seed(1010)

rf_three_train<-train(Tensile.Strength~.,data=mydata_three[,1:611],

method="rf", trControl=trainControl(method = "LGOCV", number = 100,p=0.7,savePredictions = "all", verboseIter = TRUE),

tuneGrid=expand.grid(mtry=seq(100,600,by=50)))

r5<-rf_three_train$resample$RMSE

r6<-rf_three_train$resample$Rsquared

mean(r5)

sd(r5)

mean(r6)

sd(r6)

#ntree=500, mtry=550, mean rmse=1.176,sd rmse=0.198,mean rsq=0.945,sd rsq=0.025

####MC-CV KNN 6100-6700 TS#####

set.seed(1010)

knn_three_train<-train(Tensile.Strength~.,data=mydata_three[,1:611],method="knn",

trControl=trainControl(method = "LGOCV", number = 100,verboseIter = TRUE),

tuneGrid=expand.grid(k=seq(2,15,by=1)))

ss<-knn_three_train$resample$RMSE

vv<-knn_three_train$resample$Rsquared

mean(ss)

sd(ss)

mean(vv)

sd(vv)

#MC-CV, k=5, mean rmse=0.148, rmse sd=0.029, rsq mean=0.997,sd rsq=0.0008

############PLS TS 6100-6700###########

set.seed(1010)

pls_three_train<-train(Tensile.Strength~.,data=mydata_three,

method="pls", trControl=trainControl(method = "LGOCV", number = 100,p=0.7,

savePredictions = "all", verboseIter = TRUE))

r92<-pls_three_train$resample$RMSE

r94<-pls_three_train$resample$Rsquared

mean(r92)

sd(r92)

mean(r94)

sd(r94)

#ncomp=3, mean rmse=2.452,sd rmse=0.137,mean rsq=0.0.372,sd rsq=0.076, mean nrmse=0.221

2.452/(max(mydata_three$Tensile.Strength)-min(mydata_three$Tensile.Strength))

#################PCR TS 6100-6700##########

set.seed(1010)

pcr_three_train<-train(Tensile.Strength~.,data=mydata_three,

method="pcr", trControl=trainControl(method = "LGOCV", number = 100,p=0.7,

savePredictions = "all", verboseIter = TRUE))

rmean_three<-pcr_three_train$resample$RMSE

rsq_three<-pcr_three_train$resample$Rsquared

mean(rmean_three)

sd(rmean_three)

mean(rsq_three)

sd(rsq_three)

#ncomp=3, mean rmse=3.038,sd rmse=0.0937,, mean nrmse=0.276, mean rsq=0.0428,sd rsq=0.0228

3.038/(max(mydata_three$Tensile.Strength)-min(mydata_three$Tensile.Strength))

**Prediction of Dissolution profile**

set.seed(1010)

library(readxl)

X6100_6700 <- read_excel("Images and data sets/6100-6700.xlsx")

data_three<-X6100_6700

set.seed(1010)

library(readxl)

Process_conditions <- read_excel("Process conditions.xlsx")

data_three<-merge(data_three, Process_conditions, by="ID")

###6100-6700 DS #####

set.seed(1010)

library(readxl)

Dissolution_test <- read_excel("Dissolution_test.xlsx",

sheet="6hr")

Dissolution_test<-data.frame(Dissolution_test)

data_three <- merge(data_three, Dissolution_test, by="ID")

movetolast <- function(data, move) {

data[c(setdiff(names(data), move), move)]

}

data_three <- movetolast(data_three, c("X6T", "ID", "time", "date", "timestamp"))

**############RF 6100-6700 DS 6T#######**

set.seed(1010)

data_three_6hr<-data_three[,1:611]

set.seed(1010)

RF_ds_train_one_6hr<-train(X6T~.,data=data_three_6hr,method="rf",

trControl=trainControl(method = "LGOCV", number = 100,p=0.7,verboseIter = TRUE),

tuneGrid=expand.grid(mtry=seq(350,600,by=50)))

RF_6hr_mean<-RF_ds_train_one_6hr$resample$RMSE

RF_6hr_rsq<-RF_ds_train_one_6hr$resample$Rsquared

mean(RF_6hr_mean)

sd(RF_6hr_mean)

mean(RF_6hr_rsq)

sd(RF_6hr_rsq)

**############RF 6100-6700 DS 96T#######**

set.seed(1010)

data_three_96hr<-data_three[,1:611]

set.seed(1010)

RF_ds_train_one_96hr<-train(X96T~.,data=data_three_96hr,method="rf",

trControl=trainControl(method = "LGOCV", number = 100,p=0.7,verboseIter = TRUE),

tuneGrid=expand.grid(mtry=203))

RF_96hr_mean<-RF_ds_train_one_96hr$resample$RMSE

RF_96hr_rsq<-RF_ds_train_one_96hr$resample$Rsquared

mean(RF_96hr_mean)

sd(RF_96hr_mean)

mean(RF_96hr_rsq)

sd(RF_96hr_rsq)

###### 96T MC-CV 6100-6700 rfe rf regions###

set.seed(1010)

data_three_96hr<-data_three[,1:611]

set.seed(1010)

custom<-rfeControl(functions = rfFuncs, method = "LGOCV", number = 100,p=0.7 ,verbose = TRUE)

mc_cv_three_DS_96hr<-rfe(data_three_96hr[,1:610], data_three_96hr[,611], sizes = c(10:100),

rfeControl = custom)

ds_mean_three_96hr<-mc_cv_three_DS_96hr$resample$RMSE

ds_rsq_96hr<-mc_cv_three_DS_96hr$resample$Rsquared

mean(ds_mean_three_96hr)

sd(ds_mean_three_96hr)

mean(ds_rsq_96hr)

sd(ds_rsq_96hr)

mc_cv_three_DS_96hr$bestSubset

mc_cv_three_DS_96hr$optVariables

**######KNN 96 hr######**

set.seed(1010)

knn_ds_train_2hrs<-train(X2T~.,data=data_three,method="knn",

trControl=trainControl(method = "LGOCV", number = 100,p=0.7,verboseIter = TRUE),

tuneGrid=expand.grid(k=seq(2,30,by=1)))

k22<-knn_ds_train_2hrs$resample$RMSE

k33<-knn_ds_train_2hrs$resample$Rsquared

mean(k22)

sd(k22)

mean(k33)

sd(k33)
